# Multi-omics analysis positions *DNA2* at the interface of genome integrity programs and tumor behavior in pan-cancer

**DOI:** 10.1007/s10142-026-01941-w

**Published:** 2026-06-17

**Authors:** Depanshi Pandit, Amardeep Dhillon, Sanjiban Chakrabarty, Ravindranath Sanganabasappa Bilachi

**Affiliations:** 1 Manipal Institute of Technology, Manipal Academy of Higher Education, Manipal, Karnataka 576104 India; 2 The Institute for Mental and Physical Health and Clinical Translation, School of Medicine, Deakin University, Geelong, Waurn Ponds, Victoria 3216 Australia; 3 Department of Public Health Genomics, Manipal School of Life Sciences, Manipal Academy of Higher Education, Manipal, 576104 India

**Keywords:** *DNA2*, RNA-seq, Pan-cancer, Muti-omics, Replication stress, Onco-immunology

## Abstract

*DNA2* coordinates essential maintenance processes, including cell-cycle progression; however, its aberrant activity has been implicated in cancer cell survival under oncogene-induced replication stress. To date, no comprehensive pan-cancer investigation of *DNA2* has been conducted. Employing TCGA cohorts and complementary public databases, we executed the first pan-cancer multi-omics study of *DNA2*, examining transcriptomic, genomic variations, survival, immune infiltration, single-cell functional states, protein–protein interaction enrichment, and pharmacogenomic drug-response data across tumor profiles. *DNA2* was considerably overexpressed in 17 tumors compared with matching normal tissues. In endometrial cancer, the highest frequency of genetic changes (~ 7%) was observed. Univariable analyses of survival showed that elevated *DNA2* expression was related to worse overall survival in malignancies, including adrenocortical carcinoma (HR = 17.06) and mesothelioma (HR = 2.67). Single-cell omics revealed a tumor-specific correlation between *DNA2* expression and functional annotations that encompass DNA damage, angiogenesis, and proliferation. Moreover, *DNA2* expression was consistently positively associated with regulatory T-cell subtypes, but negatively related with NK cells, and cytotoxic T cells in multiple tumors as per immune landscape profiling. According to the pharmacogenomic analysis from GDSC2 dataset, tumor cells that express higher *DNA2* are more sensitive to Tozasertib, and Daporinad. DNA2 is at the core of several interrelated modules, including flap processing, telomerase extension, and cell-cycle progression, according to the enrichment study. These findings have suggested *DNA2* as a therapeutic vulnerability in cancer, a context-dependent biomarker with implications for treatment response, prognosis, and immunity.

**Supplementary Information:**

The online version contains supplementary material available at 10.1007/s10142-026-01941-w.

## Introduction

The precise synchronization of DNA replication and repair processes is essential for maintaining genome integrity. Proteins involved in processing DNA intermediates are essential for preventing replication-associated errors. Among these, eukaryotic DNA metabolism depends critically on the evolutionarily conserved DNA replication helicase/nuclease 2 (*DNA2*/*DNA2*L/h*DNA2*). *DNA2* participates in Okazaki fragment maturation by collaborating with other processing enzymes, including RPA and FEN1, through multiple, pathway-specific mechanisms (Zheng et al. 2020). It is further involved in maintaining genomic stability by processing stalled replication forks in response to DNA synthesis stress (Zheng et al. 2020). Beyond its role in replication-associated DNA processing, *DNA2* is tightly linked to cell-cycle progression, particularly during the S and G phases, as depletion of its nuclease or helicase activity in U-2-OS cells induces terminal S/G2 arrest and checkpoint activation due to unresolved S-phase DNA structures (Jia et al. 2017; Duxin et al. 2012). *DNA2* plays a critical role in homologous recombination repair (HRR) by facilitating end resection of DNA double-strand breaks (Pawłowska et al. 2017). Apart from its nuclear and cytoplasmic localization, h*DNA2* is also found in mitochondria, where it interacts with mitochondrial DNA polymerase γ (Pol γ), supporting its critical role in mitochondrial DNA (mtDNA) replication and repair (Zheng et al. 2008, 2020; Jia et al. 2017). It also plays a key role in mitochondrial long-patch base excision repair (LP-BER), acting together with FEN1 to resolve flap structures efficiently (Zheng et al. 2008, 2020). In addition, *DNA2* interacts with TRF1 and TRF2 to localize to telomeres, where it facilitates telomere end resection, thereby contributing to telomere homeostasis (Zheng et al. 2020). While these functions are indispensable for preserving cellular equilibrium, accumulating evidence (Peng et al. 2012) suggests that cancer cells exploit *DNA2*-mediated repair pathways to tolerate oncogene-induced replication stress and sustain uncontrolled proliferation. Emerging studies across individual tumor types indicate that *DNA2* is frequently dysregulated (Hudson et al. 2021; Jia et al. 2017; Zheng et al. 2020; Folly-Kossi et al. 2023) and often overexpressed in multiple malignancies (Folly-Kossi et al. 2023; Pawłowska et al. 2017; Peng et al. 2012; Kumar et al. 2017), where its elevated activity may enhance homologous recombination repair under oncogene-induced replication stress (Peng et al. 2012), support ATR-dependent replication fork stability, particularly in TP53-mutant cancers (Folly-Kossi et al. 2023), and contribute to resistance against DNA-damaging therapies (Tammaro et al. 2015; Liu et al. 2017; Karanja et al. 2014).

The DNA damage response (DDR) pathway plays a central role in maintaining genomic stability and has been extensively studied in cancer. Key DDR components, including genes involved in homologous recombination, mismatch repair, and checkpoint signaling, have been systematically characterized in pan-cancer analyses, providing important insights into tumorigenesis and therapeutic response (Shi et al. 2024; Sokol et al. 2020; Wang et al. 2025; Liu et al. 2022; Qiu et al. 2021). In addition, a recent study examined mitochondrial DNA repair gene sets in a pan-cancer context, where *DNA2* was included as part of a broader gene panel (Dong et al. 2024). However, these analyses have largely considered *DNA2* within multi-gene frameworks, without evaluating its independent contribution in depth. Although accumulating evidence links *DNA2* to tumor progression and therapeutic response, its genomic, transcriptomic, epigenetic, and immunological landscape remains incompletely characterized. Because replication stress tolerance is a fundamental vulnerability of malignant cells, defining the pan-cancer landscape of *DNA2* is essential to link its genome-maintenance functions to disease progression and therapeutic resistance, thereby enabling biomarker-driven patient stratification and precision-targeted intervention. A comprehensive multi-omics pan-cancer analysis was therefore conducted to examine *DNA2* expression across TCGA tumors by integrating transcriptomic, genomic, epigenetic, prognostic, functional, immune, and drug-response profiling. This integrated approach delineates both shared and cancer-type–specific functions of *DNA2* and supports its evaluation as a potential biomarker and therapeutic target.

## Methodology

### Gene expression analysis across pathological stages and common cell lines

RNA sequencing–based differential expression analysis between tumor and adjacent normal tissues across TCGA cancer types was performed using the Gene_DE module of TIMER 3.0 (https://compbio.cn/timer3/) (Cui et al. 2025). Within this platform, gene expression values are represented as log₂(TPM + 1) normalized data. Differential expression was computed using the edgeR algorithm applied to raw RNA-Seq count data (Cui et al. 2025). Expression distributions were illustrated using box plots, and statistical significance was denoted according to the following thresholds: *p* < 0.05 *, *p* < 0.01**, and *p* < 0.001 ***(Cui et al. 2025).

To expand the analysis to include metastatic tissues and independent microarray datasets, the Gene Expression Comparison module of TNMplot v2 (https://tnmplot.com/analysis/) (Bartha et al. 2021) was utilized. This platform integrates gene-chip–based expression data from 33,520 samples across 3,180 studies (Bartha et al. 2021). Differences in *DNA2* expression among Normal, Tumor, and Metastatic tissues were evaluated using the non-parametric Kruskal–Wallis test (Bartha et al. 2021).

Expression profiling in in -vitro models was conducted using the Cell Line resource of the Human Protein Atlas (HPA) (https://www.proteinatlas.org/) (Jin et al. 2023). This resource provides genome-wide RNA expression data for human protein-coding genes across 1,206 human cell lines, including 1,132 cancer cell lines (Jin et al. 2023). RNA expression levels are reported as normalized transcript per million (TPM) values (Jin et al. 2023). *DNA2* expression patterns across various cancer cell line categories were assessed, and graphical representations were generated using RAWGraphs 2.0 (https://app.rawgraphs.io/) (Mauri et al. 2017).

### Univariate survival analysis

Survival analyses of *DNA2* across TCGA tumor types were performed using GEPIA3 (https://gepia3.bioinfoliu.com/) (Kang et al. 2025). For univariate survival analysis, patients were divided into high and lowexpression groups based on the median expression cutoff, with 50% of patients assigned to each group (high: 50%; low: 50%). Overall survival (OS), defined as the time from diagnosis to death from any cause, was selected as the clinical endpoint, and survival duration was measured in months. Hazard ratios (HRs), 95% confidence intervals (CIs), and corresponding p-values were obtained for each TCGA tumor type. A p-value < 0.05 was considered statistically significant.

### Gene alteration assessment

Genomic alterations of *DNA2* across human cancers were investigated using cBioPortal for Cancer Genomics (https://www.cbioportal.org/) (Gao et al. 2013). This platform integrates multidimensional cancer genomics datasets, including somatic mutations, DNA copy-number alterations (CNAs), mRNA and microRNA (miRNA) expression, DNA methylation, protein abundance, and phosphoprotein abundance (Gao et al. 2013). The analysis was performed using the TCGA Pan-Cancer Atlas studies selected through the “Quick Select” option, comprising 32 studies with a total of 10,967 samples. Cancer type–specific alteration frequencies of *DNA2* were examined to determine the distribution and prevalence of genomic alterations across tumor types. Associations between mRNA expression and copy-number alterations (mRNA vs. CNA), as well as mRNA expression and mutation type (mRNA vs. mut type), were evaluated. mRNA expression data were quantified using the RSEM (RNA-Seq by Expectation-Maximization) abundance estimation normalization method. To assess mutation distribution within functional regions of the protein, mutation mapping across *DNA2* domains was analyzed to identify the localization of somatic variants within specific structural domains.

Epigenetic alterations were further explored using MethMarkerDB (https://methmarkerdb.hzau.edu.cn/home), which integrates 724 DNA methylation biomarker genes and compiles information from PubMed-indexed publications and extensive whole-genome bisulfite sequencing (WGBS) data (Zhu et al. 2024). The Pan-cancer Differentially Methylated Region (DMR) module was employed using default parameters to evaluate *DNA2* methylation patterns across cancer types.

### Single-cell gene expression and functional states analysis

To investigate *DNA2* expression at single-cell resolution, the Single Cell module of the Human Protein Atlas (HPA) was utilized (https://www.proteinatlas.org/humanproteome/single+cell) (Karlsson et al. 2021). This resource compiles 34 independent single-cell RNA sequencing datasets sourced from publicly available repositories, including the Single Cell Expression Atlas, Human Cell Atlas, Gene Expression Omnibus (GEO), EMBL-EBI BioStudies, and Tabula Sapiens (Karlsson et al. 2021). To ensure high-quality data inclusion, only studies comprising more than 4,000 cells and exceeding 20 million sequencing reads were considered (Karlsson et al. 2021). Gene expression levels were summarized as normalized counts per million (nCPM) across annotated single-cell populations (Karlsson et al. 2021).

The functional relevance of *DNA2* at the single-cell level was further examined using the CancerSEA web platform (https://biocc.hrbmu.edu.cn/CancerSEA/home.jsp) (Yuan et al. 2019). This database assesses correlations between gene expression and 14 defined cancer-related functional states using transcriptomic data from 41,900 cancer single cells across 25 tumor types (Yuan et al. 2019). Correlation analyses were performed separately for each cancer type, focusing on datasets with a high cell count. Statistical filtering was applied with a significance threshold of *p* < 0.05 (FDR), and the correlation strength parameter was set to the “all” option. Significance levels were defined as follows: ***p ≤ 0.001, **p ≤ 0.01, and *p ≤ 0.05 (Yuan et al. 2019).

### Immune cell infiltration evaluation

Immune cell infiltration analysis for *DNA2* across TCGA cohorts was performed using the Immune Module of the GSCA (Gene Set Cancer Analysis) (Liu et al. 2023) (https://guolab.wchscu.cn/GSCA/#/). A single-gene level approach was applied to assess associations between *DNA2* and immune-related parameters, including correlations between immune cell infiltration and mRNA expression, copy number variation (CNV), and DNA methylation across TCGA tumor types. Within GSCA, the immune infiltration & mRNA expression module computes the association between gene mRNA expression and immune cell infiltration levels using Spearman’s rank correlation analysis (Liu et al. 2023). Infiltration levels for 24 immune cell populations are estimated using the ImmuCellAI algorithm integrated into the platform (Liu et al. 2023; Miao et al. 2020). Similar correlation-based analyses are provided to evaluate relationships between immune infiltration and gene CNV and methylation status. Correlations with p-values and FDR ≤ 0.05 were considered statistically significant.The resulting datasets were downloaded in Excel format and further processed in a Python environment (Bisong 2019), where heatmaps were generated to visualize the strength and direction of correlations across TCGA cancer cohorts.

### Drug sensitivity assessment

Drug sensitivity analysis for *DNA2* was performed using GEPIA3 (https://gepia3.bioinfoliu.com/) (Kang et al. 2025), which integrates TCGA transcriptomic profiles with large-scale pharmacogenomic screening datasets. The platform incorporates drug response data from CREAMMIST, Genomics of Drug Sensitivity in Cancer (GDSC1 and GDSC2), and Cancer Therapeutics Response Portal (CTRPv2.1), encompassing sensitivity information for over 1,000 therapeutic agents (Kang et al. 2025). The Cell Line Screen module under the Drug Analysis section was used to evaluate the association between *DNA2* expression and drug response across cancer types. Drug sensitivity was assessed using the half-maximal inhibitory concentration (IC50) as the response metric, and analyses were performed against classified cancer types with “unclassified” samples excluded. For the CTRPv2.1 dataset, all available cell lines were selected under the primary site category to ensure comprehensive inclusion. Correlations with p-values and adjusted p-values ≤ 0.05 were considered statistically significant for the CREAMMIST, GDSC1, and GDSC2 datasets; however, adjusted p-values were not available for the CTRPv2.1 dataset. For visualization, the top 10 drugs demonstrating the strongest sensitivity associations with *DNA2* expression levels were identified and illustrated to highlight the most relevant therapeutic candidates.

### Gene enrichment and correlation analysis

Protein–protein interaction (PPI) network construction for *DNA2* was performed using GeneMANIA (https://genemania.org/search/homo-sapiens/), which integrates datasets from Gene Expression Omnibus (GEO), BioGRID, I2D, Pathway Commons, Memorial Sloan Kettering Cancer Center, Human Protein Reference Database, HumanCyc, IntAct, MINT, NCI-Nature Pathway Interaction Database, and Reactome (Warde-Farley et al. 2010). Networks were generated based on physical interactions, co-expression, predicted interactions, co-localization, genetic interactions, pathway associations, and shared protein domains, and genes were ranked using a network-based scoring algorithm reflecting connectivity strength to the query gene (Warde-Farley et al. 2010).

The identified interactors were subsequently analyzed using the g: Profiler web platform (https://biit.cs.ut.ee/gprofiler/gost) (Kolberg et al. 2023). Functional enrichment was performed using the g: GOSt module in Homo sapiens, which conducts over-representation analysis (ORA) and calculates cumulative hypergeometric p-values to identify statistically significant terms (Kolberg et al. 2023). Detailed results were examined, and enriched terms containing *DNA2* were selected from Gene Ontology (GO) categories (GO: Molecular Function, GO: Biological Process, GO: Cellular Component) and Reactome pathway panels.

To further investigate the mitochondrial functional relevance of DNA2, an additional enrichment analysis was performed using the STRING database (https://string-db.org/) (Szklarczyk et al. 2025). DNA2 was used as the query gene, with the species restricted to *Homo sapiens* and a high-confidence interaction score threshold of 0.9. Functional enrichment analysis was conducted using GO, particularly focusing on Biological Process category. Enriched terms were ranked according to enrichment strength, and the top 20 significantly enriched biological processes were selected for interpretation. Statistical significance was determined using a false discovery rate (FDR) cutoff of ≤ 0.05.

Finally, binding partners of *DNA2* from PPI network were subjected to correlation analysis using the TNMplot platform (https://tnmplot.com/analysis/) (Bartha et al. 2021). Correlation matrix analysis was performed using RNA-Seq data from TCGA cancers, with tissue type restricted to tumor samples. Spearman correlation analysis was applied to assess the strength and direction of associations between *DNA2* and its interacting genes across TCGA tumor datasets.

## Results

### DNA2 expression patterns across pathological stages and cell line models

**Fig. 1:**
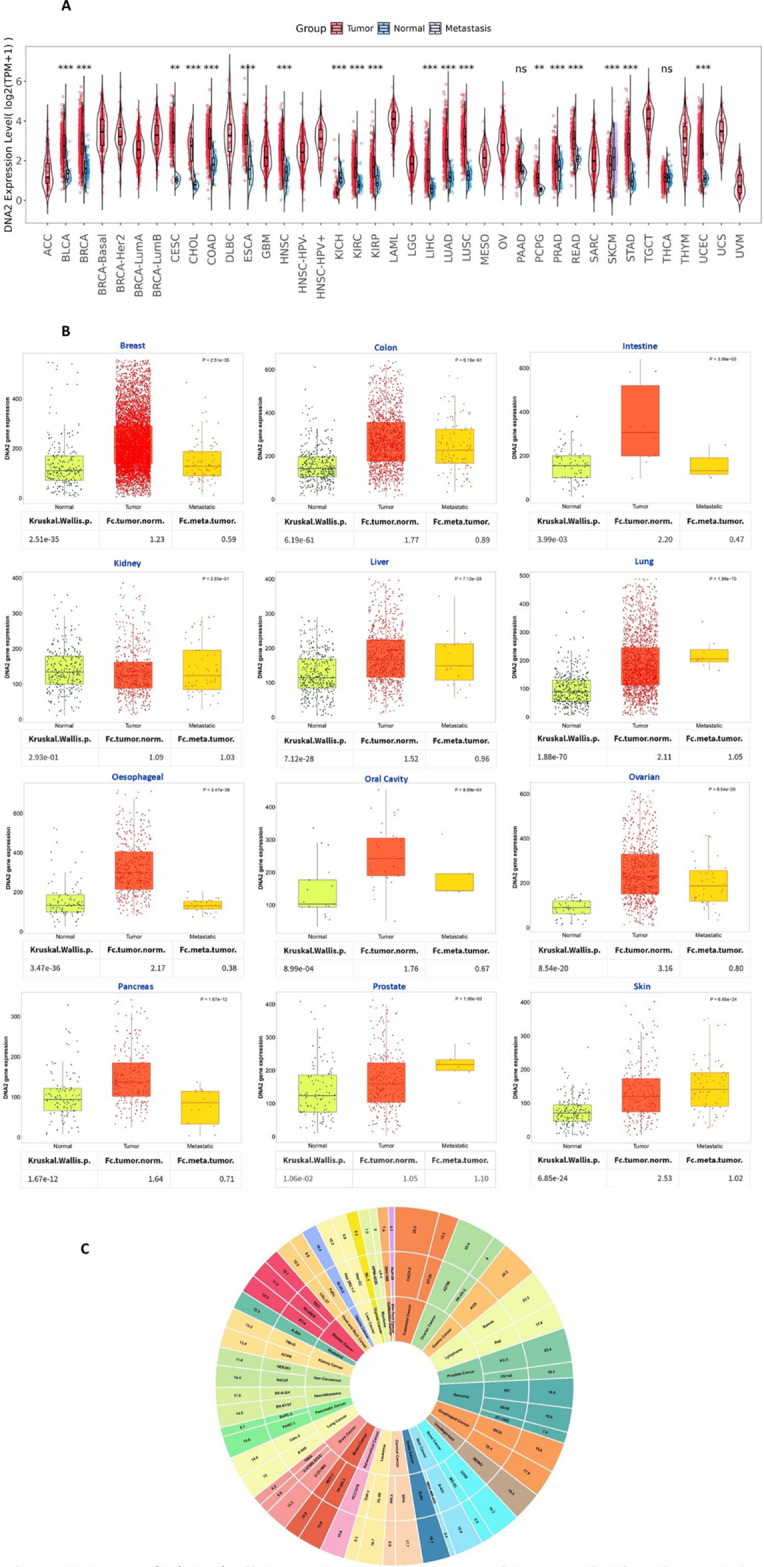
**A** *DNA2* expression (log₂(TPM + 1) profiles in TCGA tumor, normal, and metastatic tissues from TIMER 3.0. All significant values are marked as : *p* < 0.05 (*), *p* < 0.01 (**), and *p* < 0.001 (***). **B** Differential expression across pathological states in multiple organs assessed by gene-chip-based analysis using the Kruskal–Wallis test of the TNM plot tool (significance value, *p* < 0.05). **C** *DNA2* RNA expression across common cancer cell lines, quantified as normalized transcripts per million (nTPM), using data from the HPA

To evaluate *DNA2* expression across cancers, we used TIMER3 to compare tumor tissues with their adjacent normal counterparts in TCGA datasets. *DNA2* was found to be widely dysregulated across multiple cancers. Among 19 cancer types showing significant differences, 17 exhibited marked upregulation in tumors compared to normal healthy tissues, incorporating BRCA, BLCA, CESC, COAD, CHOL, ESCA, HNSC, KIRP, KIRC, LIHC, LUSC, LUAD, PRAD, PCPG, READ, STAD, and UCEC (Fig. 1A). Notably, in SKCM, *DNA2* levels were substantially higher in metastatic tissue than in the primary tumor (*p* < 0.001) (Fig. 1A). However, in the case of KICH tumor samples, the level of *DNA2* was found to be downregulated compared with non-cancerous tissues (*p* < 0.001) (Fig. 1A).

Cross-tissue gene expression analysis by TNMplot reveals significant differential expression between normal, tumor, and metastatic samples across multiple cancer types. In the majority of tissues examined, tumor samples showed a marked increase in expression compared to healthy tissues. Statistically significant differential expression was found in colon cancer, breast cancer, pancreatic cancer, small intestine cancer, esophageal cancer, lung cancer, liver cancer, ovarian cancer, oral cavity cancer, and skin cancer (Kruskal-Wallis p-values ranging from 10⁻³ to 10⁻⁷⁰) (Fig. 1B). Fold-change analysis confirmed elevated expression in primary tumors relative to normal tissues, although strongest increase was detected in ovarian (FC = 3.16), skin (FC = 2.53), intestine (FC = 2.20), lung (FC = 2.11), and oesophageal cancers (FC = 2.17) (Fig. 1B). Moderate upregulation was observed in breast, colon, pancreas, oral cavity, prostate and liver tumors (FC ~ 1.0–1.8) (Fig. 1B). In contrast, kidney samples did not show significant differences between groups (*p* = 0.293) (Fig. 1B). Metastatic samples generally displayed expression levels comparable to or slightly lower than primary tumors. In most cancer types, metastatic-to-tumor fold changes were close to 1 including oesophageal (FC = 0.38), intestine (FC = 0.47), and oral cavity cancers (FC = 0.67).

HPA RNA expression data demonstrated wide irregularity in gene expression in commonly used cell lines, with values ranging from 3.4 to 29.3 nTPM. The highest expression was observed in CACO-2 (29.3 nTPM), followed by A2780 (25.4 nTPM) and AGS (25.2 nTPM) (Fig. 1C). Several additional cell lines, including Ramos, PC-3, RD, OE33, BEWO, and U2OS, also showed relatively high expression (> 19 nTPM). A large subset of cell lines exhibited intermediate expression (~ 10–18.9 nTPM), while lower expression levels (< 8 nTPM) were detected in lines such as HT-1080, SNU-308, BxPC-3, LP-1, HuH-28, and MDA-MB-435(Fig. 1C).

### Survival impact of DNA2 expression based on univariate analysis

**Fig. 2:**
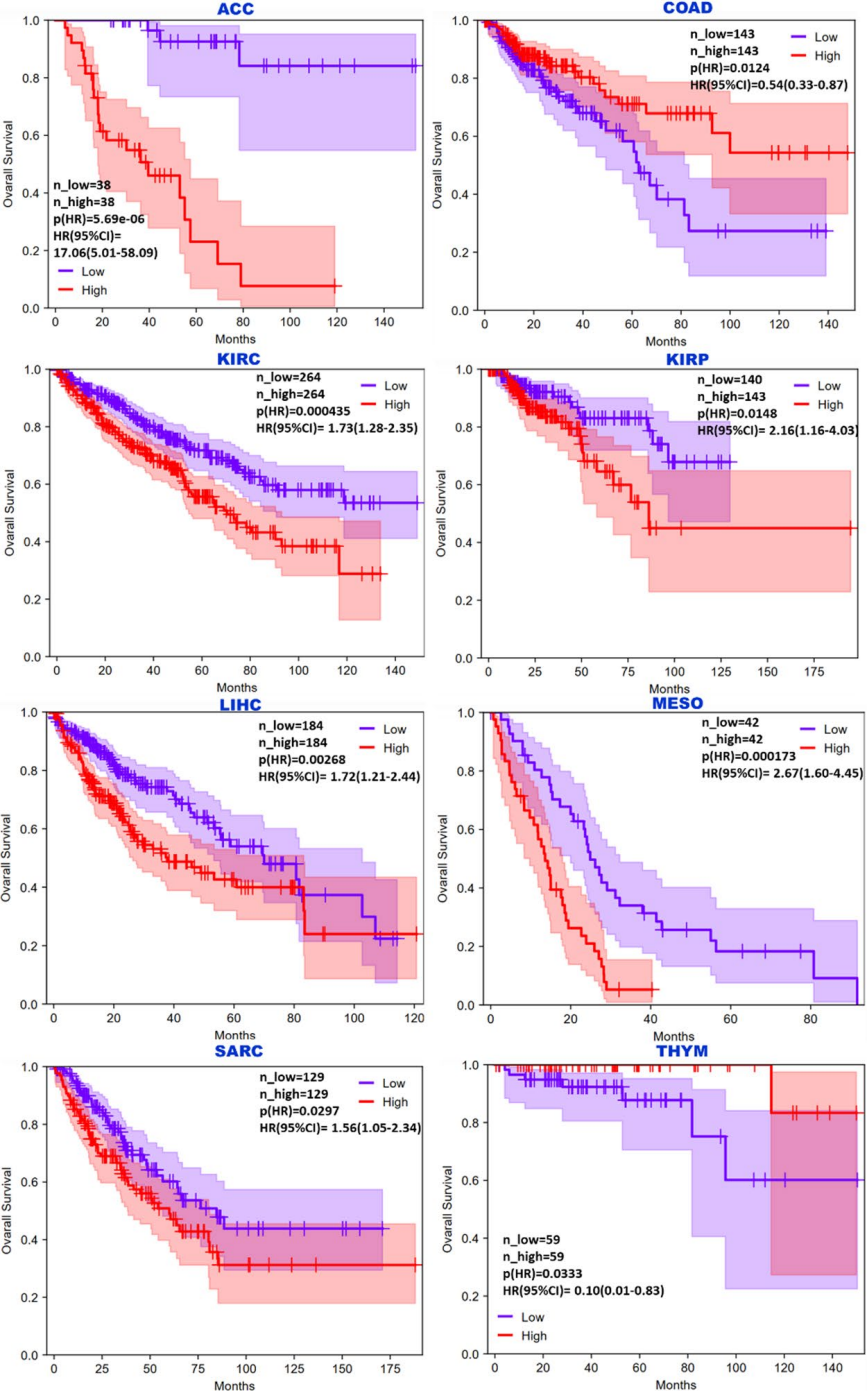
**A** Kaplan–Meier curve depicting univariate survival analysis of *DNA2* mRNA levels in patient cohorts from TCGA; only statistically significant associations are shown (*p* ≤ 0.05)

Univariate survival studies was conducted for multiple cancer cohorts in order to assess the prognostic significance of *DNA2* gene expression. High *DNA2* expression was substantially linked with adverse survival in ACC, KIRP, KIRC, LIHC, MESO, and SARC in the univariate analysis (Fig. 2A). The strongest adverse association was observed in ACC (HR = **17.06**, p = **5.69 × 10⁻⁶**) and MESO (HR = **2.67**, p = **1.73 × 10⁻⁴**), followed by KIRP (HR = **2.16**, p = **0.0148**), KIRC (HR = **1.73**, p = **4.35 × 10⁻⁴**), LIHC (HR = **1.72**, p = **0.00268**), and SARC (HR = **1.56**, p = **0.0297**). In contrast, higher *DNA2* expression was associated with improved survival in COAD (HR = **0.54**, p = **0.0124**) and THYM (HR = **0.10**, p = **0.0333**). The remaining tumor types did not retain statistical significance (supplementary 1).

### Evaluation of DNA2 gene alterations

**Fig. 3:**
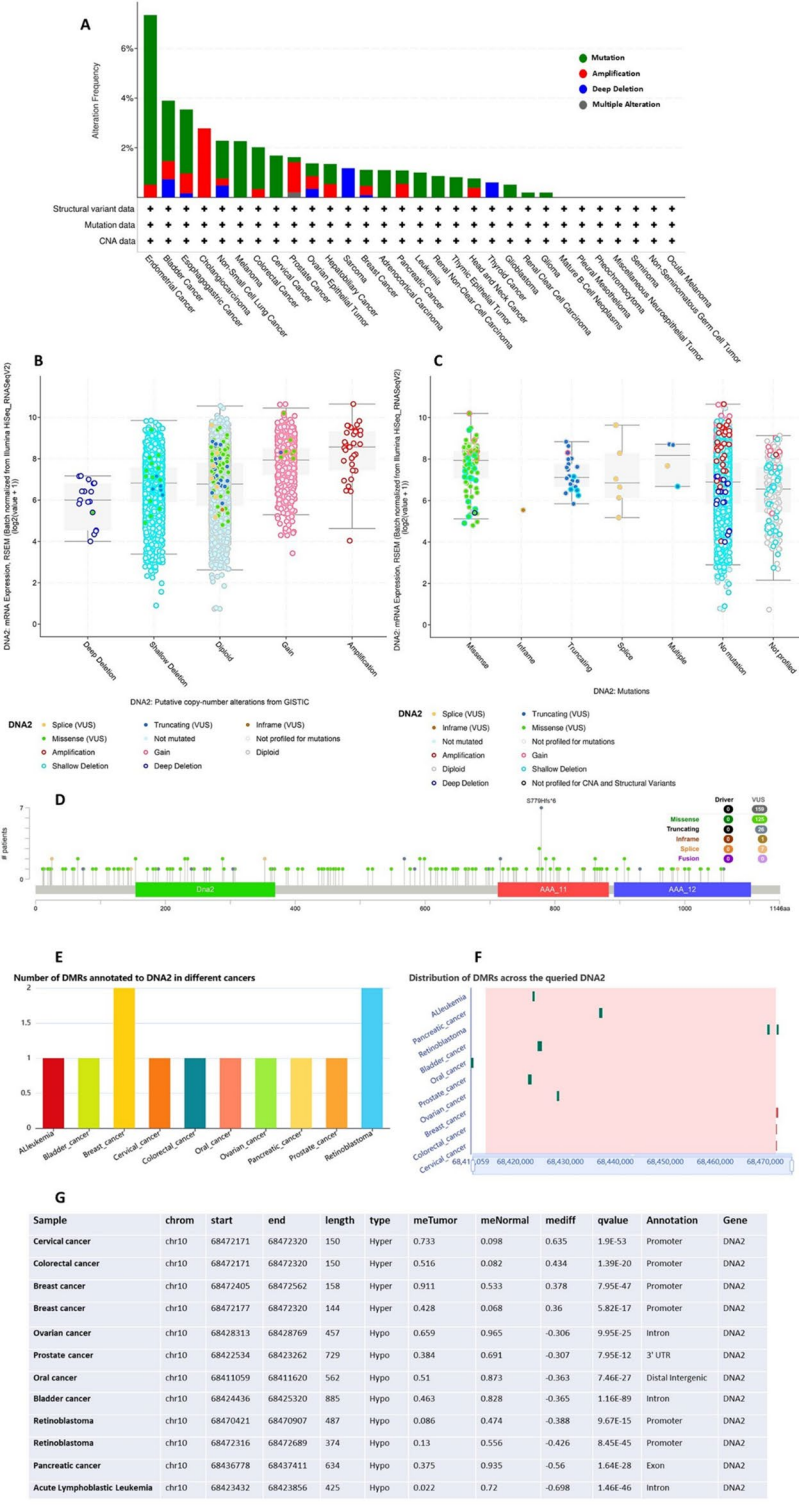
**A** Alteration frequency of *DNA2* genetic events across patient samples from the TCGA pan-cancer dataset analyzed using the cBioPortal. **B** Box plots illustrate the association between *DNA2* mRNA expression levels and copy number alterations. **C** Distribution of *DNA2* mRNA expression across different mutation categories derived from cBioPortal analysis. **D** Schematic representation of *DNA2* protein domains (amino acids 0–1146), highlighting mutation sites identified across TCGA cohorts using cBioPortal. **E**,** F&G** Number, genomic distribution, and methylation status (hypermethylated and hypomethylated) of DMRs associated with *DNA2* across pan-cancer types, annotated by genomic features, obtained from the MethMarkerDB

We assessed gene alterations to determine how frequently *DNA2* is genomically dysregulated across cancer types. Across the pan-cancer cohort, the highest incidence of *DNA2* alterations (~ 7%) was observed in endometrial cancer, which also exhibited the greatest mutation burden as depicted in Fig. 3A. Alterations in bladder and esophagogastric cancers occurred at ~ 3–4%, with mutations representing the primary event in both tumor types. In contrast, alterations in cholangiocarcinoma were mainly characterized by gene amplification (Fig. 3A).

The relationship between genomic alterations and transcriptional output was examined by comparing *DNA2* mRNA expression across copy-number and mutation categories. Samples harboring *DNA2* amplification or copy-number gain exhibited higher expression levels than diploid tumors, whereas shallow deletions were associated with reduced expression (Fig. 3B). Deep deletions showed the lowest expression overall, indicating that *DNA2* expression scales with copy-number changes (Fig. 3B). However, no distinct separation was found between tumors with missense, truncating, splice, in-frame, and multiple mutations (Fig. 3C). The distribution of *DNA2* expression across these mutation classes is very similar to that in non-mutated samples, suggesting that mutation type does not play an essential part in *DNA2* expression.

Somatic mutations in the amino acid sequence of the *DNA2* protein, as identified by the TCGA Pan-Cancer Atlas, are distributed across the entire amino acid sequence, from position 0 to 1146, with no apparent clustering in specific functional regions as illustrated in Fig. 3D. The mutation pattern was primarily represented by missense mutations (*n* = 125), along with some truncating (*n* = 26), splice-site (*n* = 7), and in-frame (*n* = 1) mutations, and no driver mutations were identified (Fig. 3D). The mutation frequency for each residue was low, although a truncating frameshift mutation at S779Hfs*6 had the highest frequency among patients (Fig. 3D).

The differential methylation analysis showed that statistically significant DMRs were found for *DNA2* in different cancer types. The majority of cancers had at least one DMR in the *DNA2* locus, with breast cancer and retinoblastoma having two DMRs, indicating that methylation alterations occur in cancer (Fig. 3E&F). Cervical, colorectal, and breast cancers had hypermethylation in the promoter region, while the majority of other cancer types, including ovarian, prostate, oral, bladder, pancreatic, retinoblastoma, and acute lymphoblastic leukemia, had hypomethylation, mainly in intronic, exonic, untranslated, and distal regions (Fig. 3G).

### Single-cell insights into DNA2 expression and functional states

**Fig. 4:**
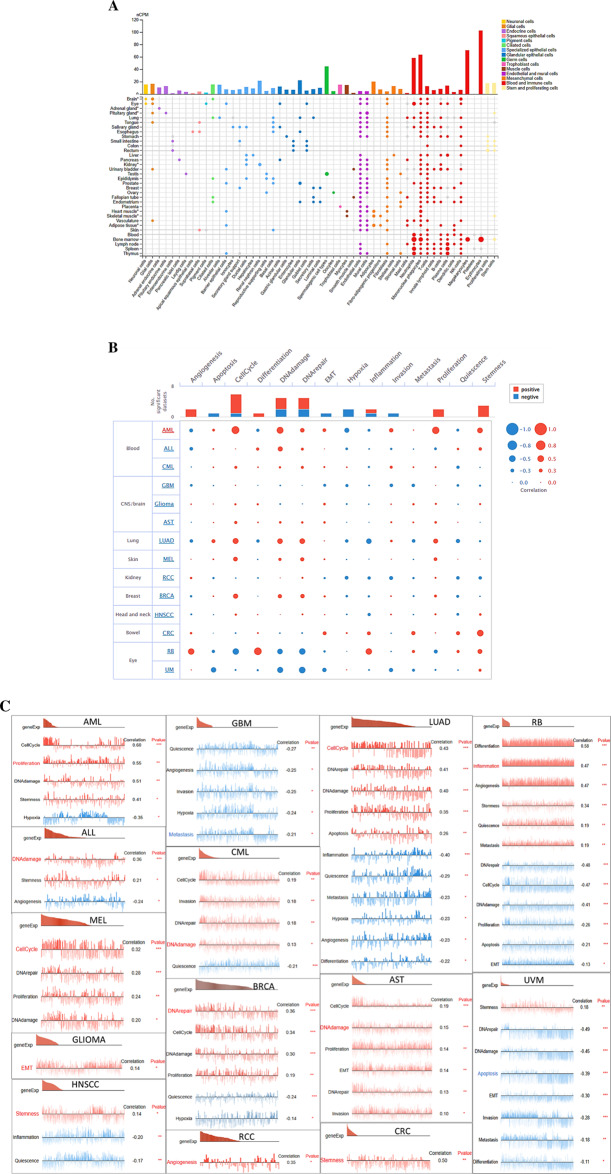
**A** *DNA2* expression at single-cell resolution across distinct cell lineages was assessed using RNA-sequencing datasets from the HPA, with expression levels reported as CPM. **B** Spearman’s rank correlation heatmaps illustrate the association between *DNA2* expression and functional annotations at the single-cell level across multiple tumor types using CancerSEA. **C** Significant functional associations FDR (*p* < 0.05) of *DNA2* expression across individual tumor types

Single-cell analysis was performed using a combination of expression data from the “Human Protein Atlas” and functional state data from “CancerSEA” to identify the distribution of *DNA2* expression and potential functional associations. The transcriptomic analysis of *DNA2* expression, measured as normalized counts per million (nCPM) in the Human Protein Atlas, revealed lineage-specific expression in tissues. Lineages of immune and hematopoietic origin showed the highest transcriptional output (> 100 nCPM), with broad distribution across lymphoid and myeloid lineages as showcased in Fig. 4A. Epithelial lineage showed low to moderate *DNA2* expression (1–23 nCPM), limited to specific tissues, whereas mesenchymal and endothelial lineages showed moderate expression (5–20 nCPM) across a wide range of tissues (Fig. 4A). Neuron and glial lineages showed specific expression in tissues of the nervous system, although at lower abundance (< 17 nCPM) (Fig. 4A). The stem/proliferative lineage showed a distinct distribution, with relatively high levels of *DNA2* expression in the gastrointestinal tract (17–18 nCPM) (Fig. 4A).

Expanding upon tumor-type-specific expression patterns, single-cell studies identified associations between *DNA2* expression and functional states that vary by context, shared across cancer types (Fig. 4B). In particular, correlation studies found that *DNA2* expression showed a moderate positive correlation with cell cycle progression, proliferation, and DNA damage, which were shared across several cancer types, including AML, BRCA, and MEL (Fig. 4C). On the other hand, it also had a moderate negative correlation that were frequently observed with quiescence, hypoxia, and metastatic signatures in GBM, and LUAD (Fig. 4C). Distinct tumor-type–specific patterns were evident, with strong positive associations with differentiation (Corr = 0.58, *p* < 0.001) and moderate associations with angiogenesis (Corr = 0.47, *p* < 0.001) and inflammation (Corr = 0.47, *p* < 0.001) in RB; weak but significant positive relationships with EMT (Corr = 0.14, *p* < 0.01) and proliferation (Corr = 0.14, *p* < 0.01) in AST; and moderate stemness-associated correlations in CRC (Corr = 0.50, *p* < 0.01) as depicted in Fig. 4C.

### Immune cell intrusion analysis in pan-cancer cohorts

**Fig. 5:**
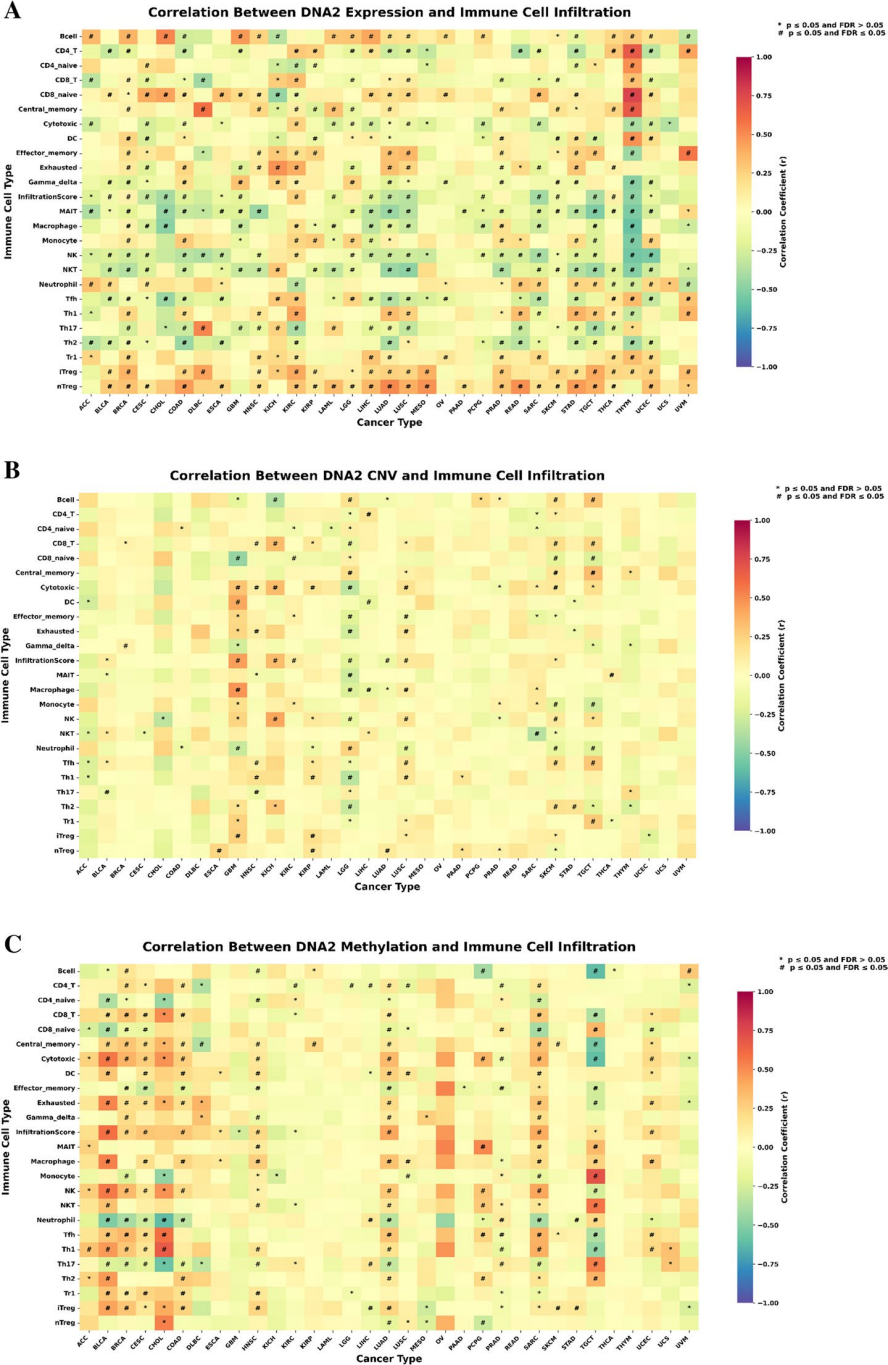
**A** Correlation heatmaps illustrate the association between *DNA2* gene expression and immune cell types across TCGA tumor cohorts, **B** the relationship between *DNA2* copy number variations and immune cell types, and **C** the association between *DNA2* methylation levels and immune cell types across cancers. All analyses were conducted using the GSCA webserver. Spearman correlation analysis was applied, and statistically significant associations are indicated at *p* ≤ 0.05and *FDR ≤ 0.05*

Using immune infiltration correlation analysis across pan-cancer, we performed a thorough assessment of the association between *DNA2* gene expression and tumor-immune microenvironment. In BRCA, HNSC, KIRC, LIHC, PRAD, and UCEC, the regulatory T-cell subtypes nTreg, iTreg, and Tr1 showed positive associations with *DNA2* expression; several of these relationships were statistically significant (FDR < 0.05) (Fig. 5A). However, in numerous solid tumors, consisting of COAD, LUAD, LUSC, THYM, etc., cytotoxic and effector immune cell types, comprising NK cells, NKT cells, cytotoxic T cells, and MAIT cells, exhibited primarily significant negative correlations (Fig. 5A). In contrast, CD8_T cells and CD4_T cells revealed a substantial positive relationship with *DNA2* expression in KIRC and THYM (Fig. 5A). In Fig. 5B, a less pronounced, heterogeneous immunological pattern was observed for *DNA2* CNV–immune correlations. In GBM, *DNA2* CNV showed significant positive associations with cytotoxic cells, dendritic cells, macrophages, and iTreg cells, whereas CD8⁺ naïve T cells and neutrophils exhibited significant negative correlations (Fig. 5B). In HNSC, SKCM, and TGCT, positive correlations were identified between *DNA2* CNV and cytotoxic and Tfh cell infiltration (Fig. 5B).

The association between immune cell intrusion and *DNA2* methylation was examined in respect to various cancer types. Numerous immune cell subsets population, comprising cytotoxic T cells, NK cells, CD8 + T cells, induced regulatory T (iTreg) cell, and Th1 cell subsets in BLCA, BRCA, CHOL, COAD, LUAD, and SARC, had favorable relationships with *DNA2* methylation (Fig. 5C). After controlling for False Discovery Rate (FDR), a substantial proportion of these correlations were found to be significant, indicating a robust association between *DNA2* methylation and immunological microenvironments in cancer tissues. However, heterogeneity by cancer type was observed, with negative associations (*p* < 0.05) reported for particular immune cell categories, such as CD8+ naïve T cells and neutrophils, in BLCA, BRCA, CESC, and LUAD (Fig. 5C).

### Drug sensitivity evaluation of DNA2 expression

**Fig. 6:**
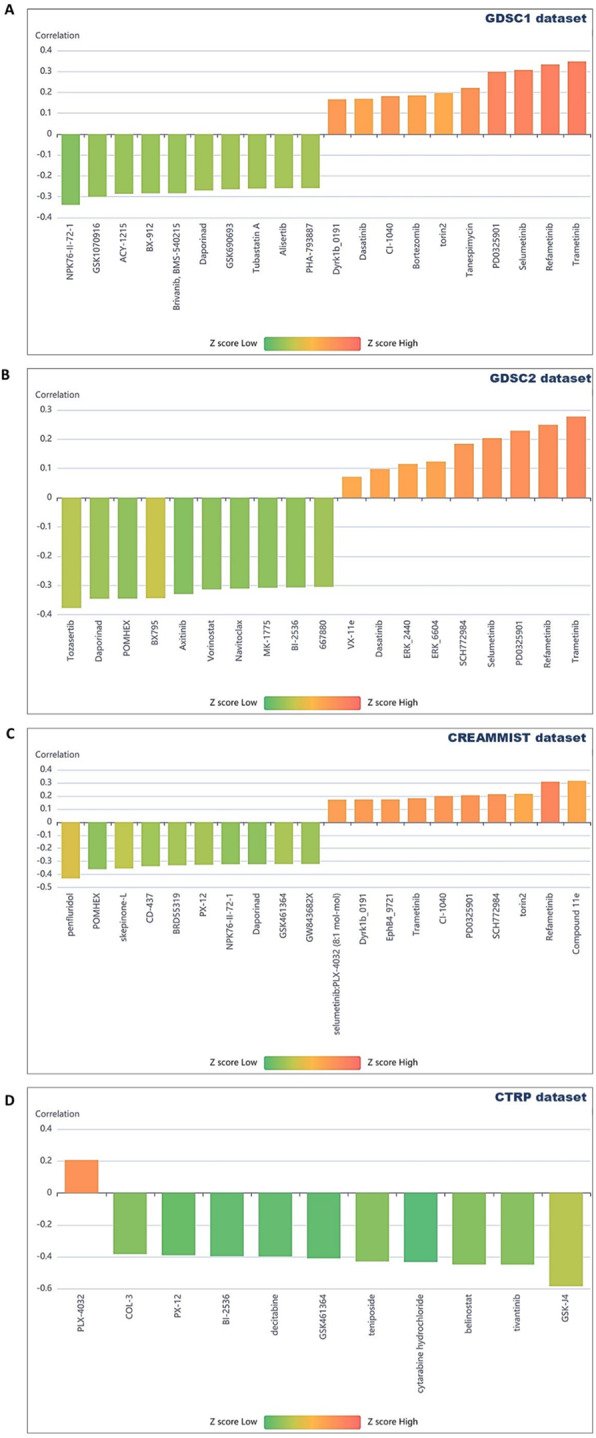
**A** Drug sensitivity analysis for *DNA2* expression using the GDSC1 dataset, **B** GDSC2 dataset, **C** CREAMMIST dataset, and **D** CTRP dataset, all analyzed via the GEPIA3 platform. Drug sensitivity was evaluated using IC50 as the response metric across pan-cancer cell lines. Spearman correlation analysis was performed for the GDSC1, GDSC2, and CREAMMIST datasets, and only drugs with *p* and *adjusted p ≤ 0.05* were included. Pearson correlation analysis was applied for the CTRP dataset (no *p* and *adjusted p values*). For each dataset, only the top 10 drugs showing significant differences in response between *DNA2* high- and low-expression groups are displayed

Given the observed expression patterns of *DNA2*, we next investigated its relationship with drug response using pharmacogenomic sensitivity datasets. Using the GDSC1 dataset (Fig. 6A), correlation analysis across cancer types demonstrated a clear bidirectional association between *DNA2* expression and drug sensitivity. The strongest significant negative correlation (highest sensitivity in tumors with high *DNA2* expression) was observed for NPK76-II-72-1 (z = -9.56, p = **2.95e-21**), followed by GSK1070916 (z = -8.18, p = **4.51e-16**), and ACY-1215 (z = -7.99, p = **2.02e-15**. In contrast, the strongest positive correlation (highest sensitivity in tumors with low *DNA2* expression) was observed for Trametinib (z = 9.64, p = **1.39e-21**), followed by Refametinib (z = 9.52, p = **4.08e-21**), and Selumetinib (z = 8.71, p = **5.35e-18**). Similarly, in Fig. 6B, analysis of the GDSC2 dataset across cancer cell lines revealed an association between *DNA2* expression and drug sensitivity. Among drugs showing higher sensitivity in tumors with high *DNA2* expression, the strongest negative correlations were observed for Tozasertib (z = -5.84, p = **7.8e-09**), followed by Daporinad (z = -7.8, p = **1.16e-14**) and POMHEX (z = -8.74, *p* = 4.98e-18). Conversely, drugs demonstrating higher sensitivity in tumors with low *DNA2* expression showed the strongest positive correlations for Trametinib (z = 7.9, p = **4.0e-15**), followed by Refametinib (z = 6.99, p = **3.48e-12**) and PD0325901 (z = 6.47, p = **1.14e-10**). Likewise, analysis of the CREAMMIST dataset (Fig. 6C) showed that high *DNA2* expression was associated with increased sensitivity to penfluridol (z = − 3.47, p = **0.000601**), POMHEX (z = − 8.84, p = **2.24e-18**), and skepinone-L (z = − 5.34, p = **1.22e-07**). However, low *DNA2* expression correlated with increased sensitivity to Compound 11e (z = 2.46, p = **0.0141**) and Refametinib (z = 8.54, p = **2.41e-17**). Finally, by utilizing the CTRP dataset across cancer cell lines (Fig. 6D), the elevated *DNA2* expression was associated with increased sensitivity to GSK-J4 (z = − 5.7916) and tivantinib (z = − 9.4122).

#### DNA2 network-based enrichment and correlation analysis

**Fig. 7:**
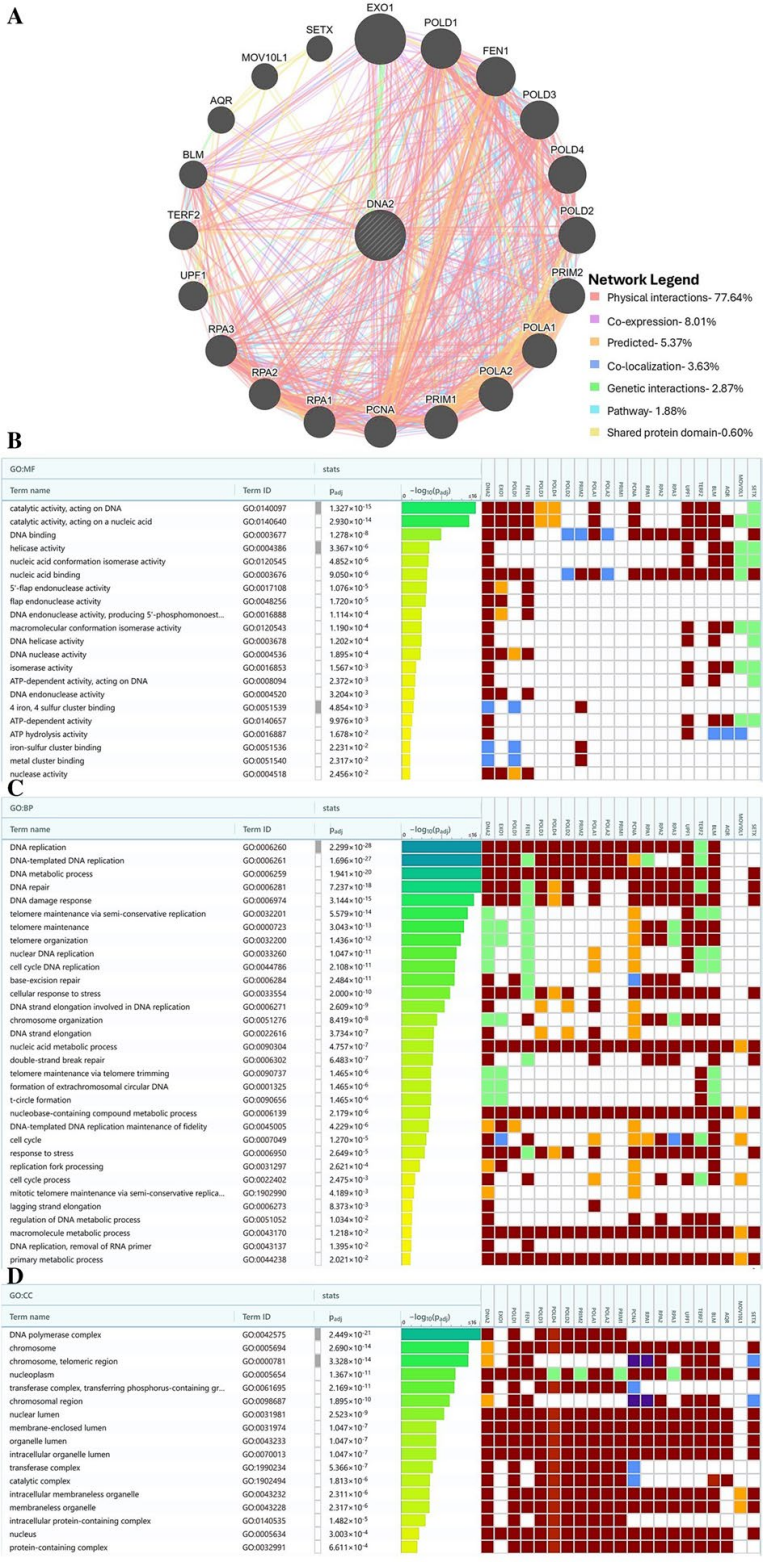

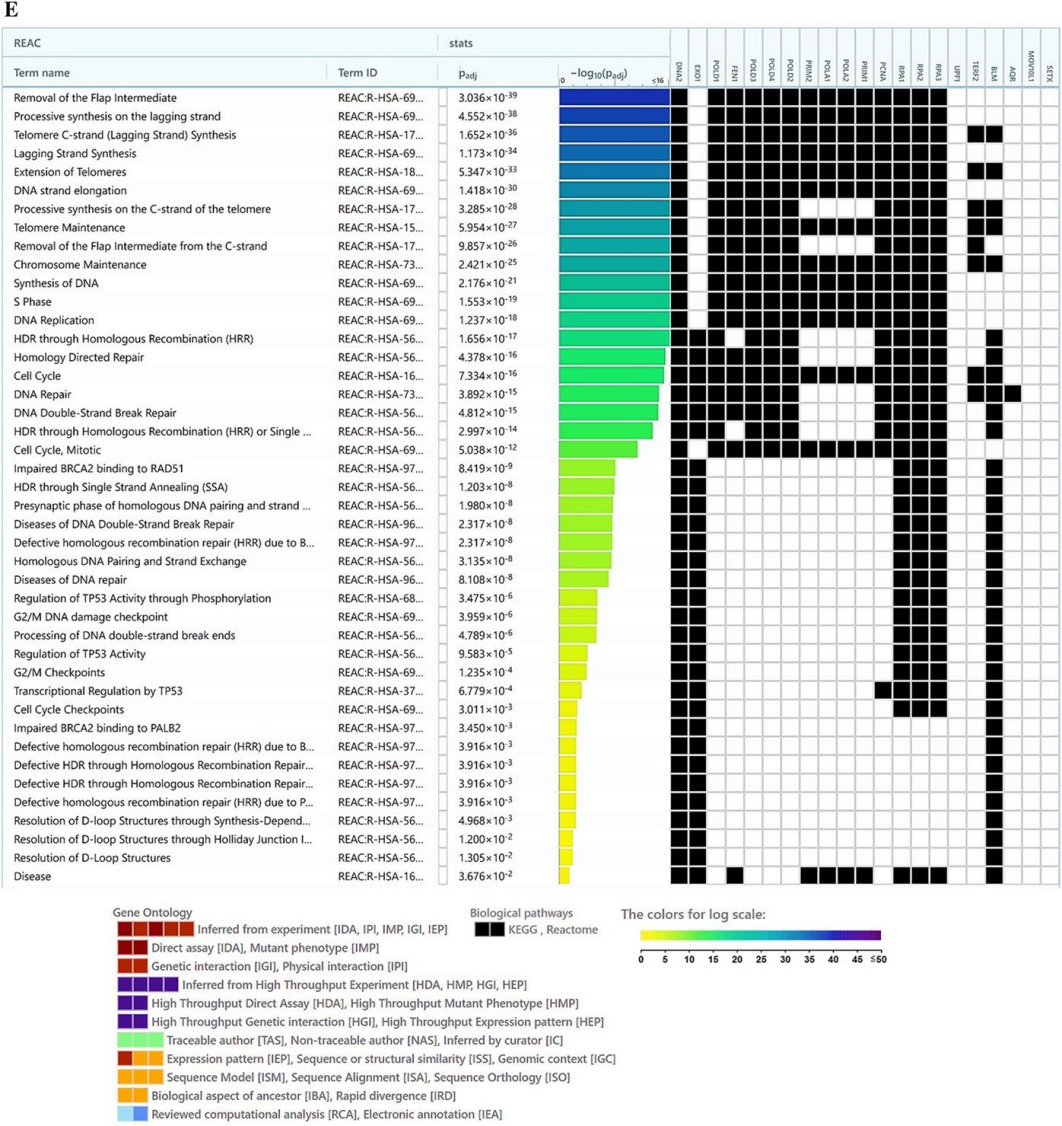

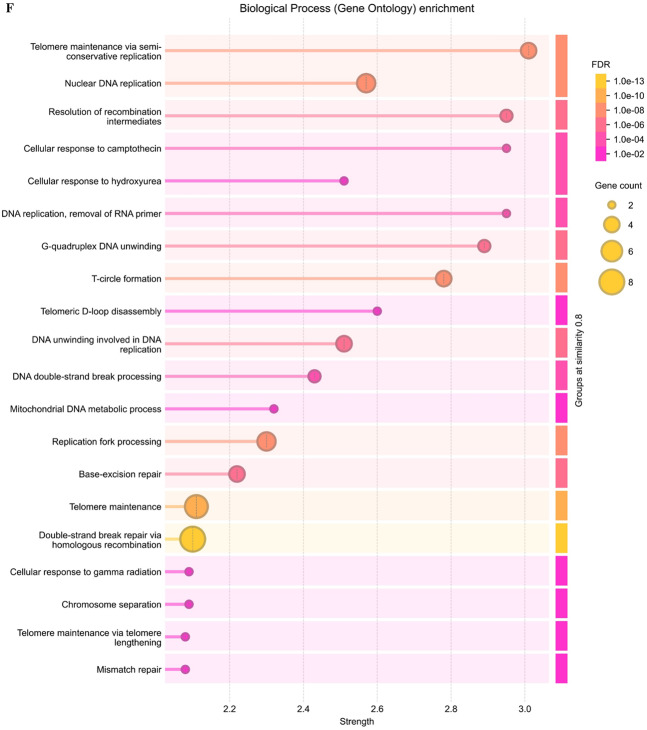
**A** Protein–protein topological interaction network for DNA2 using the GeneMANIA platform, with genes ranked according to network-based scoring and node size reflecting relative importance. **B** Functional gene set enrichment analysis of the identified DNA2 interactors was performed using g: Profiler, covering Gene Ontology categories of Molecular Function, **C** Biological Process, **D** Cellular Component, and **E** Reactome pathway enrichment, with only significantly enriched terms (*p* < 0.05) displayed. **F** STRING functional enrichment analysis showing the top 20 GO Biological Process terms ranked by strength parameter. Only significantly enriched pathways with FDR ≤ 0.05 were included

To gain mechanistic insight into the role of *DNA2* in genome maintenance, an integrative systems-level analysis encompassing interaction network topology, enrichment profiling, and cross-cancer correlation patterns was performed. The protein DNA2 has a highly linked protein–protein interaction (PPI) network as illustrated in Fig. 7A. With the exception of direct genetic interactions, which were observed only for EXO1 , the bulk of connections in the network were based on physical interactions (77.64%), whereas direct genetic relationships accounted for only 2.87% (Fig. 7A). The replication proteins EXO1, POLD1-POLD4, FEN1, PRIM1-PRIM2, PCNA, POLA1-POLA2, and the RPA proteins RPA1, RPA2, and RPA3 make up the main clusters of the protein–protein interaction network (Fig. 7A).

Within the context of GO enrichment, significant associations were observed for DNA2 and its interacting partners across multiple categories. In the molecular function domain (Fig. 7B), substantial enrichment was detected for terms such as “catalytic activity, acting on DNA”, “catalytic activity acting on nucleic acids”, and “DNA binding”. For the biological process category (Fig. 7C), highly significant enrichment was identified for processes including “DNA replication”, “DNA metabolic process”, “DNA repair”, and “telomere maintenance”. Additional substantial associations were observed with “chromosome organization”, “cell cycle”, and “cellular response to stress”. In the cellular component category (Fig. 7D), pronounced enrichment was found for terms including “DNA polymerase complex”, “chromosome”, “chromosome, telomeric region”, and “nucleoplasm”. Furthermore, pathway enrichment analysis using the Reactome (Fig. 7E) database revealed robust statistical significance for pathways such as “Removal of the Flap intermediate”, “Extension of Telomeres”, “Cell Cycle”, and “DNA Double-Strand Break Repair”. STRING functional enrichment analysis further demonstrated significant overrepresentation of pathways associated with replication stress responses, homologous recombination, and genome stability maintenance. Notably, enrichment of the term “mitochondrial DNA metabolic process” (strength = 2.32, FDR = 0.0120) suggests a potential role of DNA2 and its interacting partners in mitochondrial genome replication and repair (Fig. 7F).

**Fig. 8:**
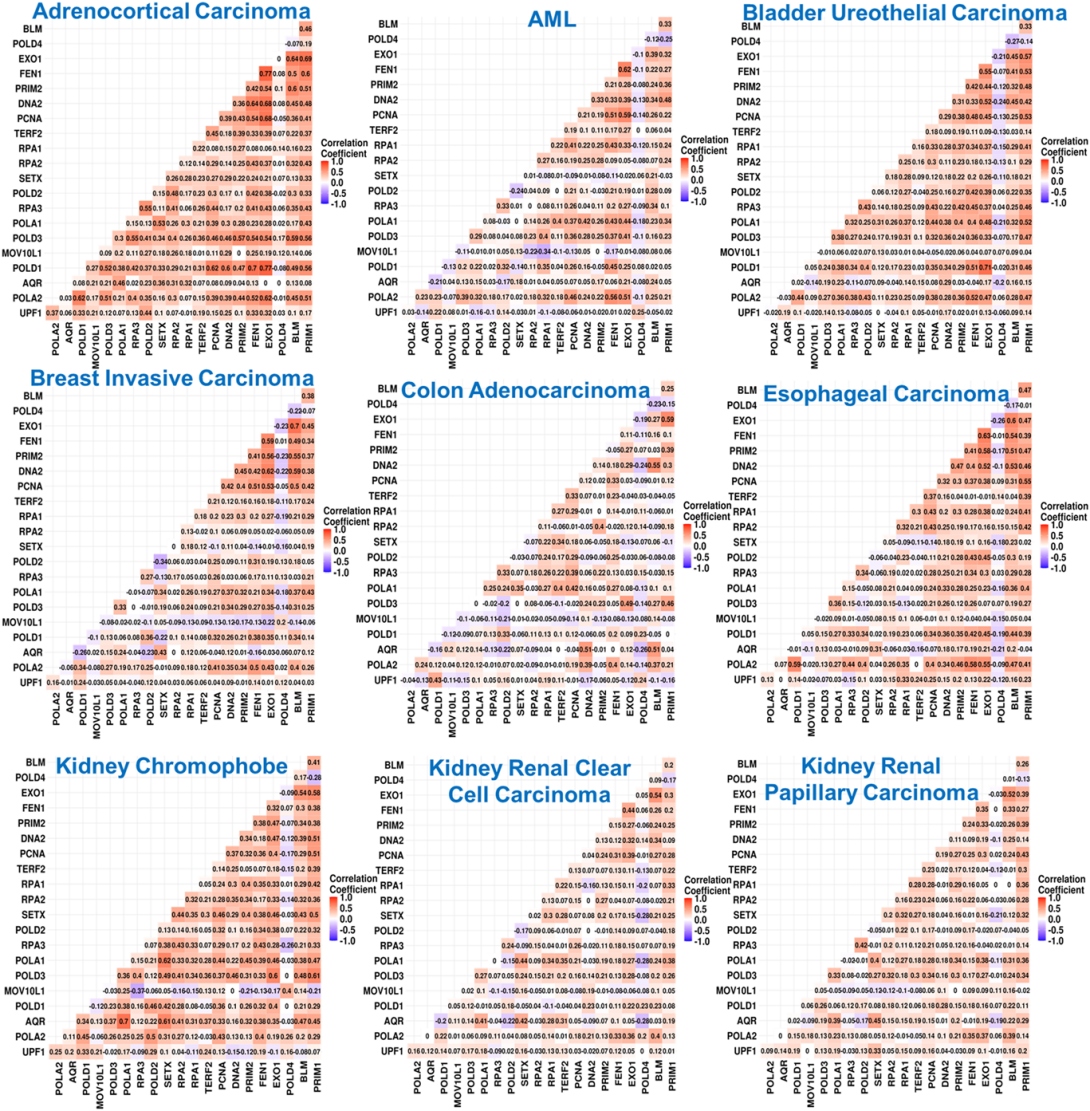

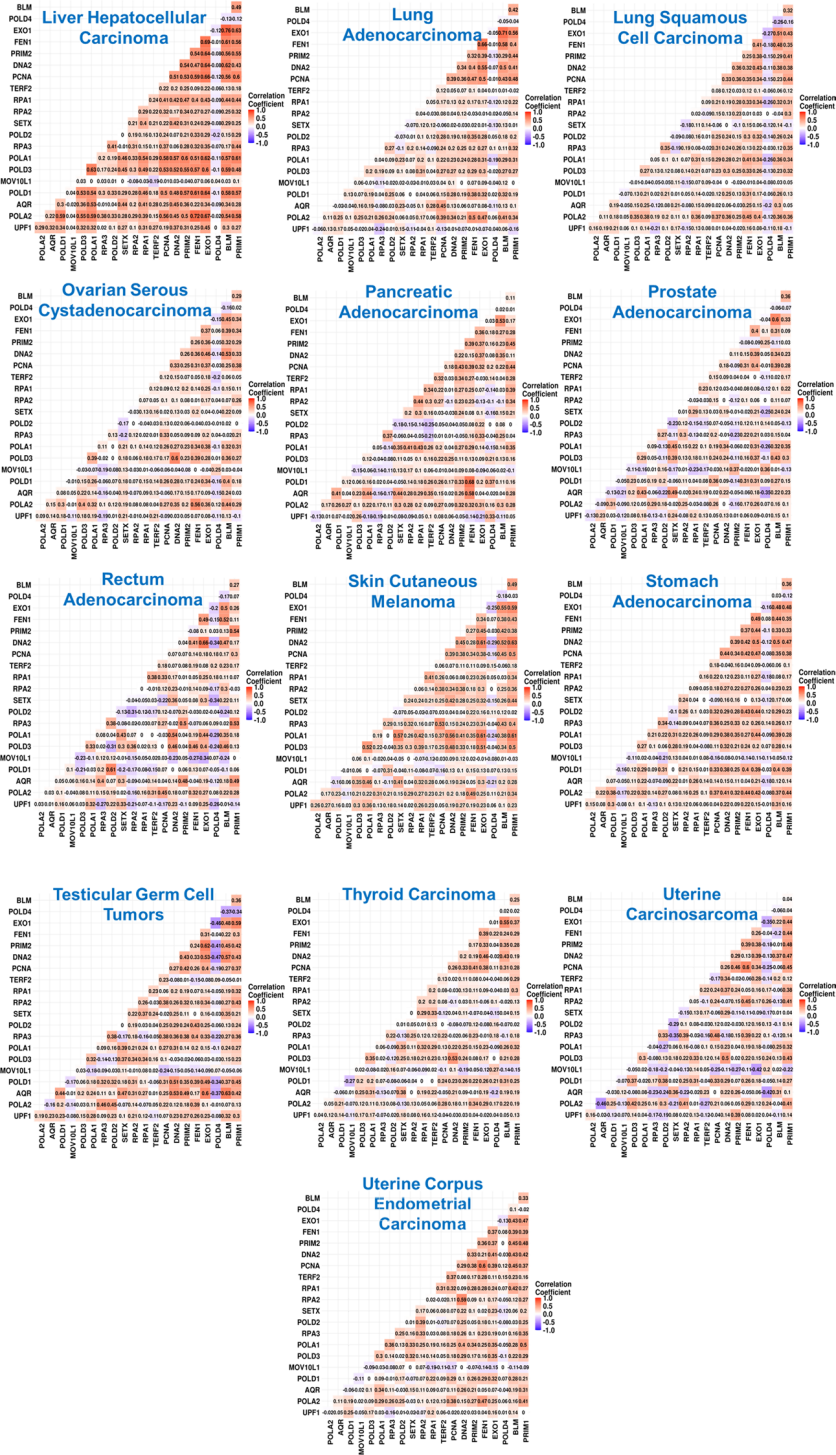
Correlation analysis between *DNA2* and its binding partners across TCGA cancer types was conducted using the TNMplot, by applying Spearman correlation analysis using RNA seq data

Correlation maps were produced to assess the connection between DNA2 gene expression and its interacting partners from the PPI network across pan-cancer datasets. In all tumor types, the correlation matrices showed predominantly positive correlations, ranging from low to moderate in strength. Notably, cancer types such as adrenocortical carcinoma, bladder urothelial carcinoma, esophageal carcinoma, kidney chromophobe, and liver hepatocellular carcinoma tended to exhibit positive correlations with DNA2 expression (Fig. 8). In cancers including ovarian serous cystadenocarcinoma, skin cutaneous melanoma, and uterine corpus endometrial carcinoma, heterogeneous correlation patterns were evident (Fig. 8). In cancers such as prostate adenocarcinoma, pancreatic adenocarcinoma, and kidney renal papillary carcinoma, comparatively weaker or neutral associations with DNA2 expression were observed (Fig. 8). Among the analyzed binding partners, POLD4 exhibited largely neutral to negative correlations with DNA2 expression across numerous cancers, including adrenocortical carcinoma (ρ = 0.08), lung squamous cell carcinoma (ρ=-0.11), liver hepatocellular carcinoma (ρ=-0.08), and testicular germ cell tumors (ρ=-0.47) as depicted in Fig. 8. Similarly, MOV10L1 showed primarily neutral to weak negative associations with DNA2 expression in numerous tumors, such as uterine corpus endometrial carcinoma (ρ = 0), breast invasive carcinoma (ρ=-0.12), and thyroid carcinoma (ρ=0.1); however, in other cancer types, weak positive correlations were observed, including adrenocortical carcinoma(ρ = 0.29), kidney renal clear cell carcinoma (ρ = 0.19), and prostate adenocarcinoma (ρ = 0.14), indicating context-dependent variability in these DNA2-associated relationships (Fig. 8).

## Discussion

Targeting replication stress–related vulnerabilities is a key focus in cancer research, as tumor cells depend on genome-maintenance pathways to support rapid growth and tolerate DNA damage. *DNA2*, a multifunctional helicase/nuclease involved in DNA replication, repair, telomere processing, and mitochondrial genome stability, promotes tumor cell survival, but its broader molecular and clinical roles across cancers remain unclear. Here, we conducted a comprehensive multi-omics analysis across diverse cancer types to characterize *DNA2* dysregulation and its biological impact.

Elevated *DNA2* expression was observed across 17 tumor types (Fig. 1A), suggesting involvement in proliferative gene expression programs linked to gene duplication and cell-cycle progression, consistent with studies showing that increased *DNA2* expression promotes proliferative cellular behavior (Kumar et al. 2017; Wang et al. 2021). Previous reports have also documented *DNA2* overexpression in breast, pancreatic, endometrial, and colorectal malignancies (Han et al. 2021; Wang et al. 2023; Lu et al. 2020), with increased levels detected in tumor tissues and cell line models. Cross-tissue analyses further indicated tumor-associated upregulation, with the largest fold changes observed in lung, esophageal, ovarian, and skin cancers characterized by high proliferative turnover (Fig. 1B), suggesting that *DNA2* upregulation may occur early in tumorigenesis and persist during progression. Heterogeneous expression across cell line models as showcased in Fig. 1C reflects biological variability in replication dynamics and DNA repair pathway utilization and highlights the importance of model selection in experimental studies targeting *DNA2*; this is exemplified by higher expression in colorectal, ovarian, and gastric cell lines compared with lower levels in pancreatic, and hematologic models (Fig. 1C). Nevertheless, limited multi-level characterization of *DNA2* expression across experimental systems may limit cross-platform comparability and interpretation, underscoring the need for further investigation.

Survival analyses demonstrated that elevated *DNA2* expression is associated with adverse clinical outcomes in multiple malignancies (Fig. 2A), supporting its potential role as a prognostic biomarker. Mechanistic studies provide a biological context for these clinical observations. Given *DNA2*’s established role in DNA replication and repair, its overexpression may enhance tumor cell tolerance to replication stress and genotoxic insults, thereby promoting aggressive behavior and therapeutic resistance. In multiple myeloma, *DNA2* was identified through CRISPR/Cas9 screening as a critical mitochondrial DNA repair protein required to overcome antisense oligonucleotide induced DNA damage targeting ILF2 and to sustain metabolic adaptation following DNA damage activation (Thongon et al. 2024). Clinically, elevated *DNA2* expression was associated with chromosome 1q21 amplification and poorer overall survival in treated multiple myeloma patients, further linking *DNA2* activity to inferior therapeutic outcomes (Thongon et al. 2024). Although we did not observe a statistically significant association in ovarian cancer within our TCGA cohort, prior transcriptomic analyses have reported that elevated *DNA2* expression correlates with advanced stage, higher grade, chemotherapy resistance, and worse overall and disease-free survival in epithelial ovarian cancer (Folly-Kossi et al. 2023). These discrepancies may reflect differences in cohort composition, molecular subtype distribution, treatment stratification, sample size, or analytic methodology. Collectively, this variation highlights the context-dependent and tumor-specific prognostic role of *DNA2*, suggesting that its clinical impact may depend on underlying biological and therapeutic contexts. A limitation of the present study’s survival analysis is that GEPIA3 does not support comprehensive adjustment for important clinical confounders such as age, tumor stage, TNM classification, or treatment status across cancer types. Therefore, the prognostic associations observed for *DNA2* require validation in clinically adjusted multivariable studies.

In this study, we show that changes in *DNA2* occur at low to moderate frequencies across a variety of cancer types and are mediated by a range of complementary mechanisms, supporting a context-dependent, rather than conventional, role for a cancer driver gene. The high alteration rate in endometrial cancer is consistent with its elevated mutational burden. In contrast, mutation-predominant events in bladder and esophagogastric cancers and amplifications in cholangiocarcinoma (Fig. 3A) suggest tumor-specific modes of *DNA2* deregulation. Copy-number status closely correlates with mRNA expression as illustrated in Fig. 3B, indicating that gene dosage is a major determinant of *DNA2* transcription. In contrast, the absence of expression differences among mutation subtypes and the dispersed mutation pattern (Fig. 3C) suggest limited selection for specific functional variants. Most *DNA2* mutations were missense and lacked clear hotspots (Fig. 3D), further supporting the likelihood that many alterations represent passenger events, although modest effects on DNA replication and repair cannot be excluded. Notably, truncating variants in *DNA2* have been implicated in “mt DNA depletion syndrome” (Sun et al. 2022) and primordial dwarfism (Shaheen et al. 2014). Additionally, pathogenic mutations were demonstrated to affect the enzyme activities of the helicase and nuclease domains, thus contributing to the instability of the mitochondrial DNA in myopathy (Ronchi et al. 2013). These findings indicate that loss-of-function mutations can have significant biological consequences. Functional studies in estrogen-dependent cancers further demonstrate that mutations affecting helicase and nuclease domains can impair the enzymatic activity of *DNA2* and DNA repair capacity (Strauss et al. 2014). In parallel, widespread DNA methylation changes suggest that epigenetic regulation may represent an additional layer of tumor-specific *DNA2* modulation. Differential methylation analysis in our study identified recurrent *DNA2*-associated regions across multiple cancers, with promoter hypermethylation observed in cervical, colorectal, and breast cancers, and hypomethylation detected in several other tumor types, including oral and ovarian cancers (Fig. 3E, F&G). Given these observed methylation patterns, promoter-associated methylation may influence transcriptional output (Smith et al. 2020). In contrast, methylation elsewhere in the genome may reflect broader chromatin remodeling associated with genomic instability (Bhattacharjee et al. 2016). Further experimental and integrative analyses will be required to determine whether these methylation changes directly regulate *DNA2* expression or represent secondary consequences of tumor-associated epigenomic dysregulation.

The heterogeneity of cancer cells has been proven by several investigations on single cells, underscoring the significance of sensitive and targeted diagnostic and treatment strategies (Wu et al. 2021; Fan et al. 2025; Zhang et al. 2023; Hu et al. 2025). Significant data on the microenvironment, cancer stem cell populations, and tumor architecture could be obtained through lineage-resolved, state-specific transcriptional profiling enabled by single-cell analysis (Pfisterer et al. 2021). Leveraging single-cell expression profiles with functional state annotations, we explored the distribution of *DNA2* cell types and functional relationships across different tissues and cancer types. According to our examinations, *DNA2* expression is widely distributed over an array of cell lineages (Fig. 4A); however, the levels of expression clearly vary depending on the lineage. In particular, we see that immunological and hematopoietic populations exhibit significant transcriptional activity, whereas mesenchymal, endothelial, epithelial, and neural populations exhibit more moderate or tissue-restricted expression of *DNA2*. *DNA2* abundance occurred in either cycling or replication-competent phases in these lineages. Thus, lineage identity is not as powerful a predictor of *DNA2* expression as any proliferative condition. Functional correlation studies revealed significant context-dependent effects across tumor types as showcased in Fig. 4B, which is consistent with this explanation. *DNA2* expression is favorably correlated with programs related to cell cycle, proliferation, and DNA damage in AML, BRCA, and MEL (Fig. 4C). This aligns with *DNA2*’s known functions in DNA replication and repair. Furthermore, instead of acting as an independent driver of these programs, the data implies that *DNA2* may represent a surrogate marker of cell-cycle participation in these malignancies. On the other hand, as opposed to direct inhibition, the moderately negative relationships with quiescence, hypoxia, and metastasis were observed in GBM and LUAD and with inflammation-related signatures in LUAD. (Fig. 4C), probably reflecting changes in the composition of cellular states. Cell-cycle arrest and suppression of replication associated transcriptional programs occur because of hypoxia (Hubbi et al. 2015), while inflammatory signaling can enhance stress-responsive or senescence-like states with reduced proliferative capacity (Saito et al. 2024; Han et al. 2024). Since *DNA2* preferentially associates with cells in the cell cycle, it is most simply explained that hypoxia or inflammation does not repress *DNA2* but that *DNA2* is reduced because of enrichment in non-cycling or stressed cells in these contexts. Because the transcriptional programs associated with quiescence, inflammation and stress can overlap with senescence signatures (Colucci et al. 2025; Muñoz-Espín et al. 2014), correlations involving these states should be interpreted cautiously. Collectively, these findings imply that rather than sharing a uniform oncogenic or tumor-suppressive role, *DNA2*-associated transcriptional programs are determined by tumor type, lineage identity, and cellular state.

The tumor microenvironment (TME) plays a central function in cancer progression and response to therapy (De Visser et al. 2023). It has also been proposed that control of DNA replication and repair is associated with the heterogeneity seen in the TME (Yadav et al. 2025). Although *DNA2* has an essential position in the governance of the genome, there has been a lack of evaluation of the immunological-related functions of *DNA2*. In this study, a pan-cancer overview of *DNA2* immune correlations has been presented by combining expression, copy number, and methylation data of diverse tumor types. Across malignancies, the immunological component of cancer has been consistently and heterogeneously correlated with *DNA2* expression levels. Elevated *DNA2* expression was frequently related with increased intrusion of immunosuppressive regulatory T-cell subsets and reduced representation of cytotoxic and innate immune populations, particularly in solid tumors, while associations with CD4⁺ and CD8⁺ T cells varied by cancer type (Fig. 5A). Because the balance between CD8⁺ and CD4⁺ T cells is crucial for effective tumor suppression (Ostroumov et al. 2018), these findings indicate that *DNA2* may regulate this balance in a cancer-type–specific manner. Together, these patterns indicate that *DNA2* expression may reflect underlying differences in tumor immune contexture and suggest that *DNA2* marks tumors with diminished antitumor immune activity rather than functioning as a direct regulator of immune responses. Our findings are supported by recent multi-omics studies showing that genome maintenance–associated genes may also influence the tumor immune microenvironment. For example, an integrative analysis of the RAD21-PON1 axis in hepatocellular carcinoma demonstrated that chromatin-associated regulators can affect immune infiltration patterns and patient prognosis (Liu et al. 2025). Similarly, our results indicate that DNA2 expression is associated with distinct immune infiltration profiles across multiple cancers, suggesting a potential link between replication stress related pathways and tumor immune regulation. In contrast, *DNA2* copy number variation showed generally weak and inconsistent associations with immune infiltration as depicted in Fig. 5B, suggesting that structural alterations in *DNA2* have a limited impact on shaping immune composition at the pan-cancer level. *DNA2* methylation, however, exhibited more consistent positive correlations with cytotoxic and Th1-associated immune populations across multiple malignancies (Fig. 5C). This observation suggests that epigenetic regulation of *DNA2* may influence immune activation states and that *DNA2* methylation may serve as a marker of altered replication stress or genomic instability that permits enhanced immune engagement. On a mechanistic level, the data are consistent with the sole existing research linking *DNA2* to the immune response pathway, which demonstrated that the stability of *DNA2* is tightly regulated by CDK12-mediated phosphorylation and ubiquitin-mediated degradation pathways (Sun et al. 2025). Loss of CDK12 activity results in aberrant stabilization of *DNA2*, increased replication stress, increased levels of cytoplasmic double-stranded DNA, and initiation of the cGAS/STING pathway and innate immunity (Sun et al. 2025). In this context, overexpression of *DNA2* might counteract the buildup of cytosolic DNA and the stimulation of innate immunity, leading to a tumor microenvironment with immunosuppressive properties. On the contrary, impaired *DNA2* activity or epigenetic silencing might augment innate immune activation through the induction of DNA leakage caused by replication stress, which might explain the increased levels of cytotoxic immune cells found in tumors with increased levels of *DNA2* methylation. As immune infiltration was inferred using ImmuCellAI using GSCA platform from bulk TCGA transcriptomic data, the results may still be influenced by tumor purity and tumor microenvironment heterogeneity inherent to bulk sequencing approaches.

It is crucial to examine the association between *DNA2* expression and drug response to uncover therapeutically actionable vulnerabilities and address the long-standing issue of drug resistance in cancer. Changes in *DNA2* expression may reconfigure tumor reliance on specific signaling, metabolic, and cell-cycle pathways, thereby influencing treatment response, as *DNA2* influences the replication stress response, DNA end resection, and genome maintenance. Across GDSC1, GDSC2, CREAMMIST, and CTRP datasets, a consistent bidirectional pattern was observed (Fig. 6A, B, C, & D). Increased sensitivity to NPK76-II-72-1, Tozasertib, and other drugs that target metabolic and epigenetic pathways, such POMHEX and GSK-J4, was significantly linked with high *DNA2* expression. The increased sensitivity of *DNA2*-high tumors to Tozasertib and Daporinad as per GDSC2 may reflect a broader dependency on pathways that maintain genomic stability and metabolic adaptation under replication stress. *DNA2* is a critical nuclease/helicase involved in Okazaki fragment processing, stalled replication fork recovery, and DNA repair, and its elevated expression likely represents an adaptive response to heightened replication stress and chromosomal instability (CIN) in rapidly proliferating tumors. Aurora kinases regulate mitotic fidelity and chromosome segregation(Chung et al. 2025); therefore, inhibition by Tozasertib (Chung et al. 2025; Vats et al. 2025) may overwhelm *DNA2*-high cells that are already highly reliant on coordinated replication stress resolution and mitotic checkpoint control, ultimately inducing mitotic catastrophe and apoptosis. Similarly, Daporinad-mediated inhibition of NAMPT depletes intracellular NAD+ levels, potentially impairing the activity of NAD-dependent enzymes such as PARPs and sirtuins that are essential for DNA repair and chromatin maintenance (Mogol et al. 2024; Hasmann et al. 2003). Because DNA2-high tumors may exhibit increased dependence on DNA repair pathways to tolerate persistent genomic stress, disruption of NAD+ metabolism could further compromise repair capacity and cellular survival. Collectively, these findings suggest that elevated *DNA2* expression may define a therapeutically vulnerable state characterized by co-dependence on replication stress response, mitotic regulation, and metabolic pathways supporting DNA repair. Conversely, greater susceptibility to the MEK pathway inhibitors Trametinib and Refametinib were strongly associated with decreased *DNA2* expression. This suggests that *DNA2* expression defines tumors along a spectrum of distinct states, based on their relative sensitivities to resolving replication stress versus cell proliferation pathways. Additional research supports the idea that *DNA2* is a promising therapeutic target. To cope with replication stress, cancer cells have been shown in earlier research to overexpress *DNA2*. A homozygous deletion of *DNA2* increases the sensitivity of cells lacking *DNA2* to ionizing radiation and camptothecin (CPT) (Liu et al. 2016). According to Liu et al. (2016), 4-hydroxy-8-nitroquinoline-3-carboxylic acid (C5) is a small-molecule inhibitor of *DNA2* that selectively targets its helicase domain, thereby preventing its nuclease and ATPase activities. DNA end resection, the resumption of stopped replication forks, and homologous recombination are all functionally inhibited by C5, especially in settings when fork protection is lacking, such as BRCA2 absence (Liu et al. 2016). Importantly, pharmaceutical suppression of *DNA2* mimics genetic depletion, increases CPT sensitivity in cancer cells, and works in concert with PARP inhibitors (Liu et al. 2016). The pharmacogenomic data and findings support *DNA2* as a druggable target and predictive biomarker. Our results suggest that mechanisms that alleviate replication stress may play a major role in *DNA2*-high cancers. For these cancers, such dependency suggests that *DNA2*-targeted or combination approaches will be helpful. *DNA2*-low cancers, on the other hand, show clear treatment vulnerabilities. Therefore, a sensible plan to increase treatment efficacy and maybe delay the establishment of acquired resistance is to combine *DNA2* expression stratification with targeted intervention.

Thus, a holistic, integrative strategy that takes a network perspective is necessary to determine whether *DNA2* is a bona fide genome maintenance component or merely a peripheral nuclease. The preponderance of physical interactions supported by edges and the presence of dense clusters of proteins that interact with *DNA2*, including replication proteins (Fig. 7A), are indicative of the stable association of *DNA2* with the replication fork processing apparatus and Okazaki fragment maturation machinery (Jia et al. 2017; Hudson et al. 2021). The network architecture of DNA2 is reminiscent of essential replisome-associated proteins rather than auxiliary nucleases. Thus, although physical connections are ubiquitous, DNA2 exhibits few direct genetic interactions, restricted to EXO1 (Fig. 7A), indicative of a specialized and non-redundant functional relationship in DNA end resection during double-strand break repair (Zhu et al. 2008; Mojumdar et al. 2024). The paucity of direct genetic interactions indicates that *DNA2* is a component of a unique enzymatic reaction structurally coordinated with other nucleases but not genetically supported by them. The functional coherence of the network is further supported by Gene Ontology enrichment studies (Fig. 7B, C, &D) that link DNA2 interactors to “DNA replication”, “DNA repair”, “telomere maintenance”, and “DNA polymerase complex” organization. The Reactome enrichment (Fig. 7E) of the “Removal of the Flap Intermediate” and “DNA Double-Strand Break Repair” pathways support the physical interaction of DNA2 with FEN1 and polymerase delta subunits, thereby supporting the mechanistic function of DNA2 in flap processing and maintaining genome stability (Burgers 2011; Pawłowska et al. 2017). Additionally, enrichment of the “mitochondrial DNA metabolic process” (Fig. 7F) term suggests that the functional role of DNA2 may extend beyond nuclear genome maintenance to include mitochondrial DNA replication and repair. Supporting this observation, a previous study demonstrated that human DNA2 localizes to mitochondria, interacts with mitochondrial DNA polymerase γ, and participates in RNA primer removal and long-patch base excision repair, thereby contributing to mitochondrial genome stability (Zheng et al. 2008). Collectively, these findings suggest a broader role for DNA2 in coordinating cellular responses to replication stress and DNA damage across both nuclear and mitochondrial compartments. Expression correlation studies as illustrated in Fig. 8 identify tumor-type-specific regulation of the *DNA2* network. Positive correlation values in adrenocortical carcinoma and liver hepatocellular carcinoma indicate the coordinated upregulation of replication and repair machinery in response to heightened replication stress, whereas unique regulation of POLD4 is indicated by ρ values of -0.08 and − 0.47 in liver hepatocellular carcinoma and testicular germ cell tumors, suggesting selective regulatory divergence within the same complex. The same is true for MOV10L1, with weak negative and positive correlation values of -0.12 and 0.19 in breast carcinoma and renal clear cell carcinoma, respectively. *DNA2* is associated with cancers in which EXO1 expression is known to impact prognosis, with a *p-value *< 0.001, thereby supporting the clinical significance (Liu et al. 2025). All of the results point to a model in which *DNA2* functions as part of the replication/repair machinery, both physically integrated and transcriptionally coordinated, yet with regulatory flexibility, especially across tumor types.

Using TCGA data and the multi-omics pipeline, our work contributes to the seminal study that illuminates the role of *DNA2* in pan-cancer, providing an extensive overview of its molecular, clinical, and functional importance. Nevertheless, some limitations persist, including sample size variability across cancer types, the need for cross-platform verification, and the need for mechanistic verification of the immune component and its potential therapeutic implications. Although our integrative analyses revealed significant correlations between *DNA2* expression and multiple oncogenic, immunological, and pharmacogenomic features, these findings remain primarily correlative in nature and should therefore be interpreted cautiously. Consequently, the conclusions of this study should be regarded as hypothesis-generating rather than definitive evidence of causality. Future experimental investigations using cellular and animal models, along with validation in independent clinical cohorts, will be essential to confirm the functional role of *DNA2* in tumor progression, replication stress regulation, immune modulation, and therapeutic response. Additionally, the present study did not investigate upstream post-transcriptional regulatory mechanisms governing DNA2 expression. In addition to post-transcriptional regulation, transcriptional regulatory mechanisms may also contribute to DNA2 dysregulation across cancers. Given DNA2’s role in replication stress and genome maintenance, its expression may be coordinated by transcription factor networks involved in cell-cycle control and DNA repair. Motif-based approaches such as MotifHub (Liu et al. 2024) may help identify trans-acting regulatory motifs and upstream transcriptional programs associated with DNA2 expression. Integrating such analyses with transcriptomic and epigenomic datasets could further clarify the regulatory mechanisms underlying DNA2 overexpression in cancer. To the best of our knowledge, limited literature is currently available regarding ceRNA-mediated regulation of DNA2 across cancers. Future studies integrating computational prediction approaches with in -vitro experimental validation may help identify upstream regulatory molecules influencing *DNA2* expression and further clarify the molecular basis of its prognostic significance. In this context, recently reported ceRNA-based integrative frameworks in stomach adenocarcinoma, which combined differentially expressed lncRNAs, miRNAs, and mRNAs to construct prognostic regulatory networks and risk-scoring models (Liu et al. 2024), may provide a useful strategy for future exploration of DNA2-associated regulatory mechanisms across cancers. Detailed studies of single-cell and lineage-specific research, real-time survival outcomes, molecular epigenetics, and the preclinical validation of *DNA2*-targeted treatments, with or without replication-stress-inducing drugs, are potential future research avenues.

## Conclusion

Cancer cells may exploit *DNA2* as a non-oncogene dependency to withstand replication stress. Upregulated *DNA2* expression across cancers correlates with worse clinical prognosis, a complex immune landscape, and context-dependent functional roles at the single-cell level. Higher *DNA2* levels correlate with particular drug sensitivities, suggesting therapeutic relevance. In conclusion, *DNA2* might be a therapeutic target that capitalizes on tumor reliance on the resolution of replication stress pathways, as well as a promising biomarker for prognosis and prediction.

## Supplementary Information

Below is the link to the electronic supplementary material.

Supplementary Material 1 (DOCX 21.0 KB)

Supplementary Material 2 (XLSX 149 KB)

Supplementary Material 3 (XLSX 104 KB)

## Data Availability

The data used in this study are publicly available and were derived from web-based servers and tools as outlined in the Methodology section. All datasets were accessed through the respective platforms, and no restricted data sources were utilized. Relevant links to these platforms have been provided in the Methodology to ensure transparency and reproducibility of the results.
