# Correction to: Multi-omics analysis positions DNA2 at the interface of genome integrity programs and tumor behavior in pan-cancer

**DOI:** 10.1007/s10142-026-01962-5

**Published:** 2026-07-24

**Authors:** Depanshi Pandit, Amardeep Dhillon, Sanjiban Chakrabarty, Ravindranath Sanganabasappa Bilachi

**Affiliations:** 1 Manipal Institute of Technology, Manipal Academy of Higher Education, Manipal, Karnataka 576104 India; 2 The Institute for Mental and Physical Health and Clinical Translation, School of Medicine, Deakin University, Geelong, Waurn Ponds, Victoria, 3216 Australia; 3 Department of Public Health Genomics, Manipal School of Life Sciences, Manipal Academy of Higher Education, Manipal, 576104 India

**Correction to: Functional & Integrative Genomics**

10.1007/s10142-026-01941-w

In the original version of this manuscript, Figures 4, 7, and 8 have discrepancies between the Online PDF and SpringerLink. The HTML version contains the correct and complete figures.

The correct Figures are shown below.

Incorrect Figure 4
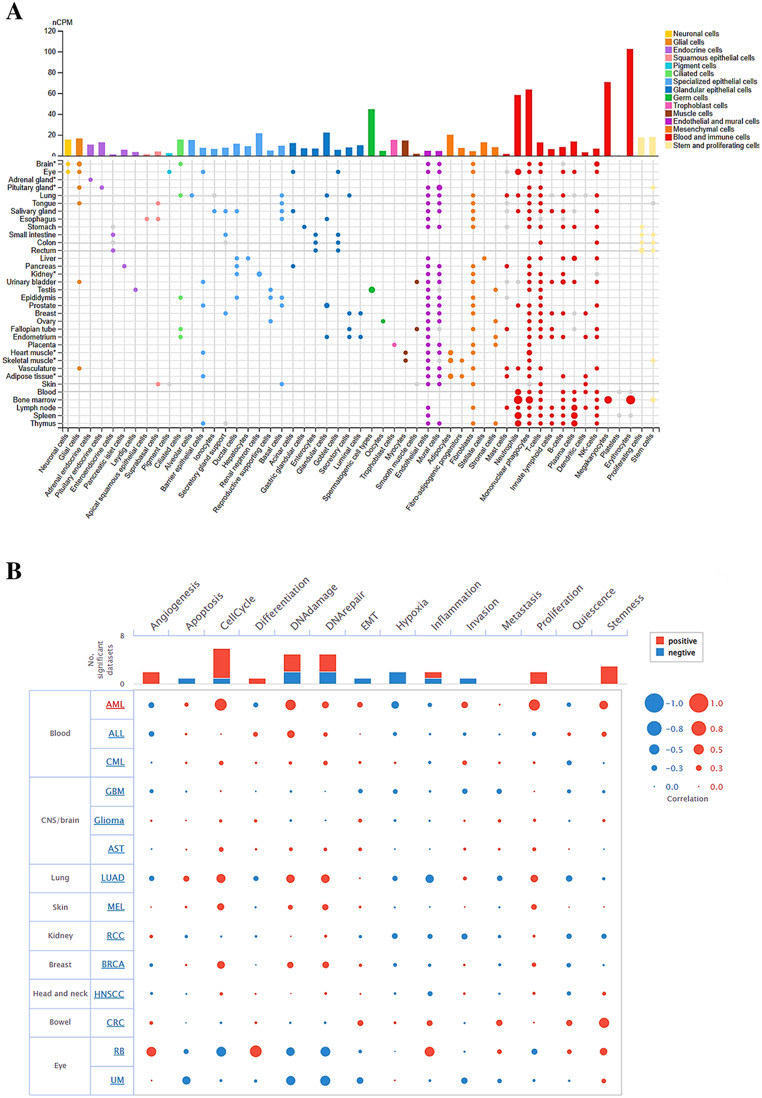


Correct Figure 4
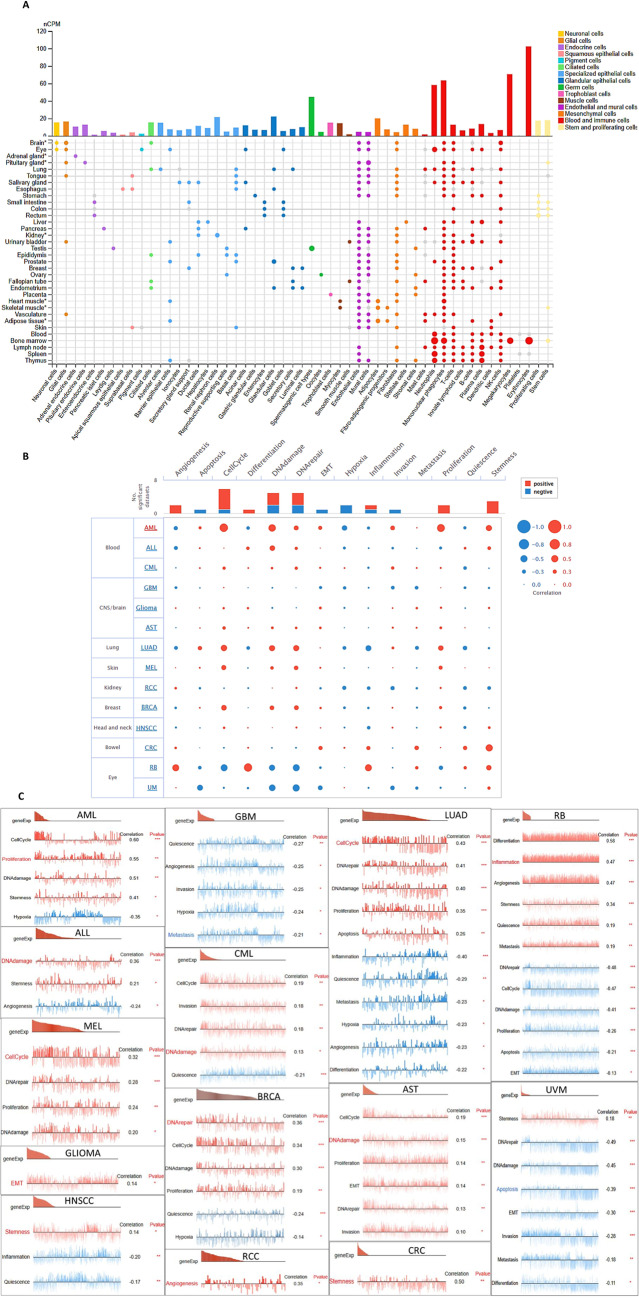


Incorrect Figure 7
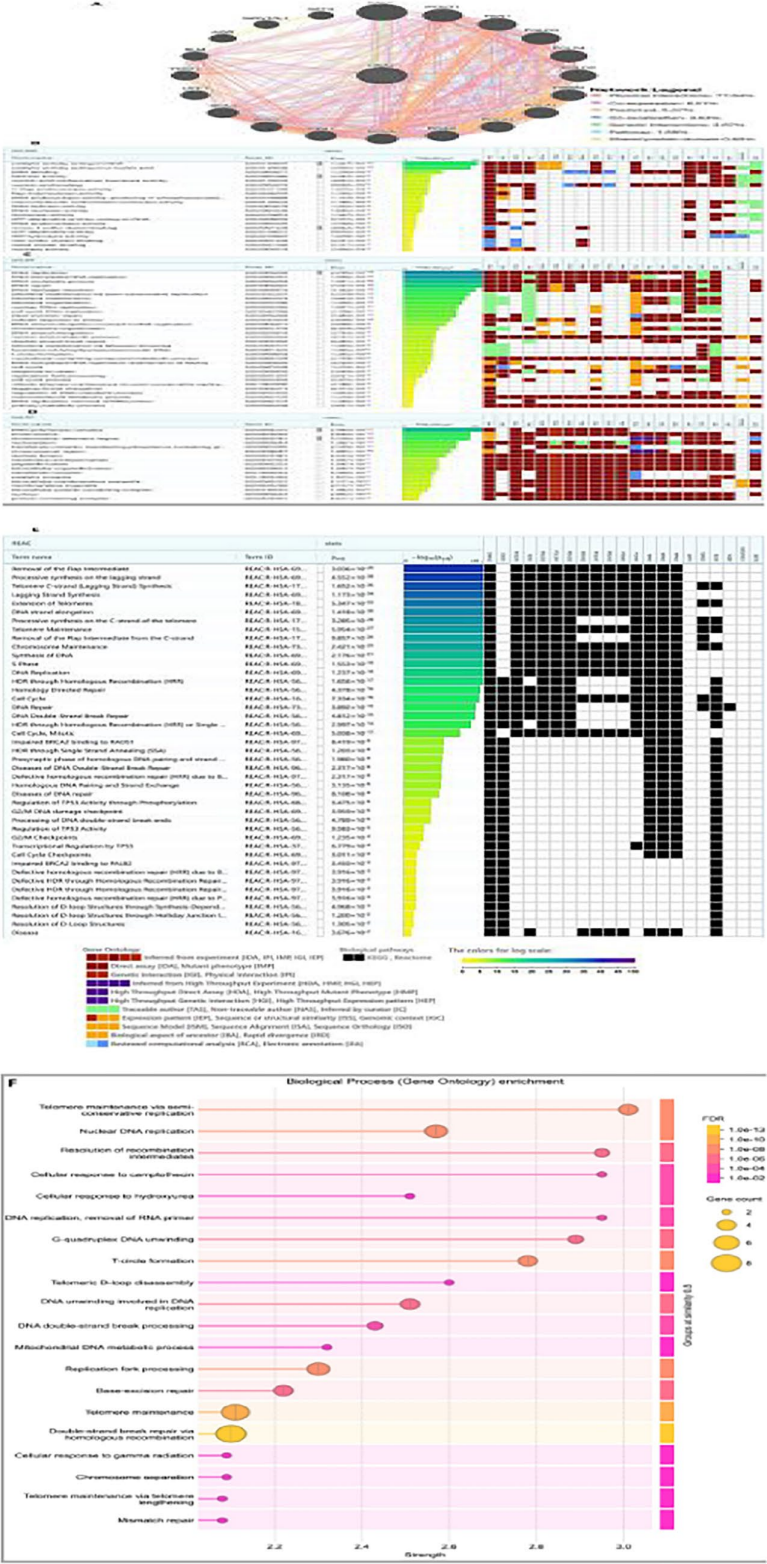


Correct Figure 7
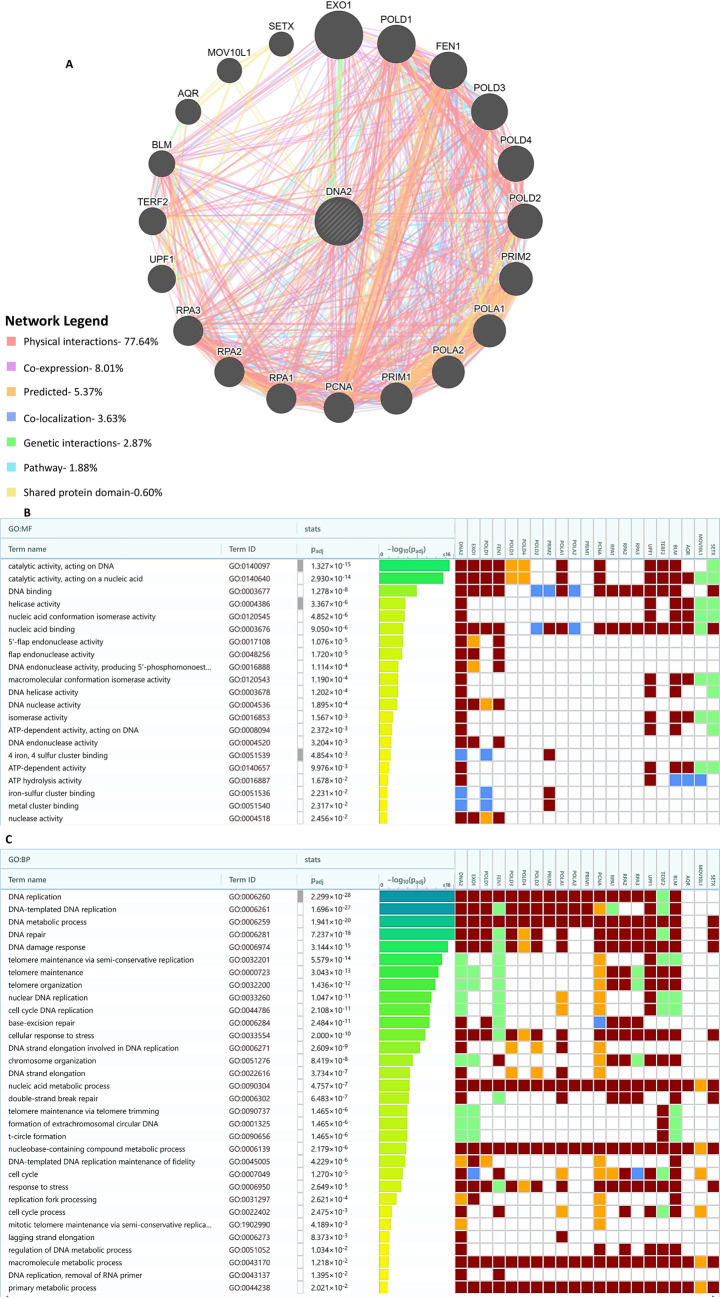

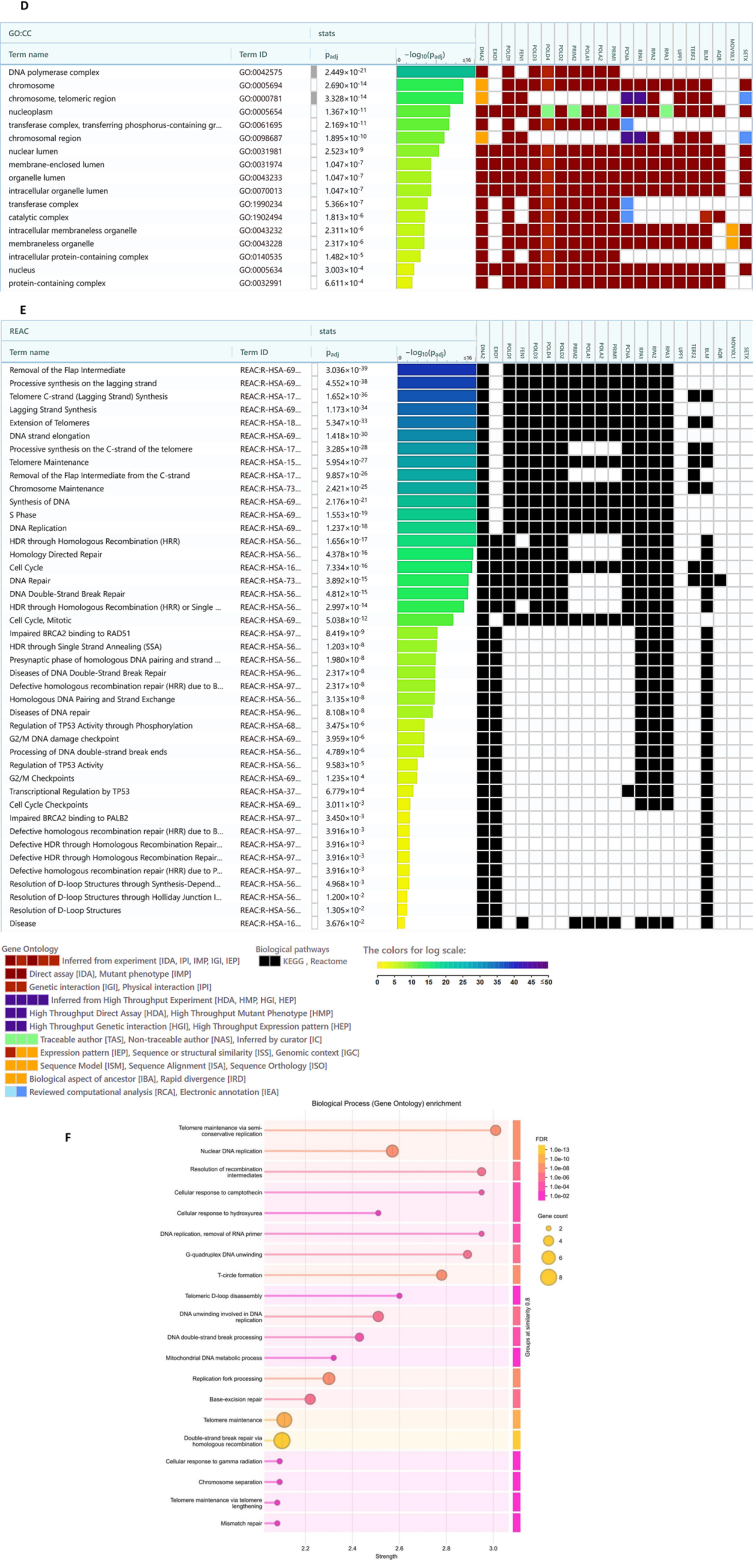


Incorrect Figure 8
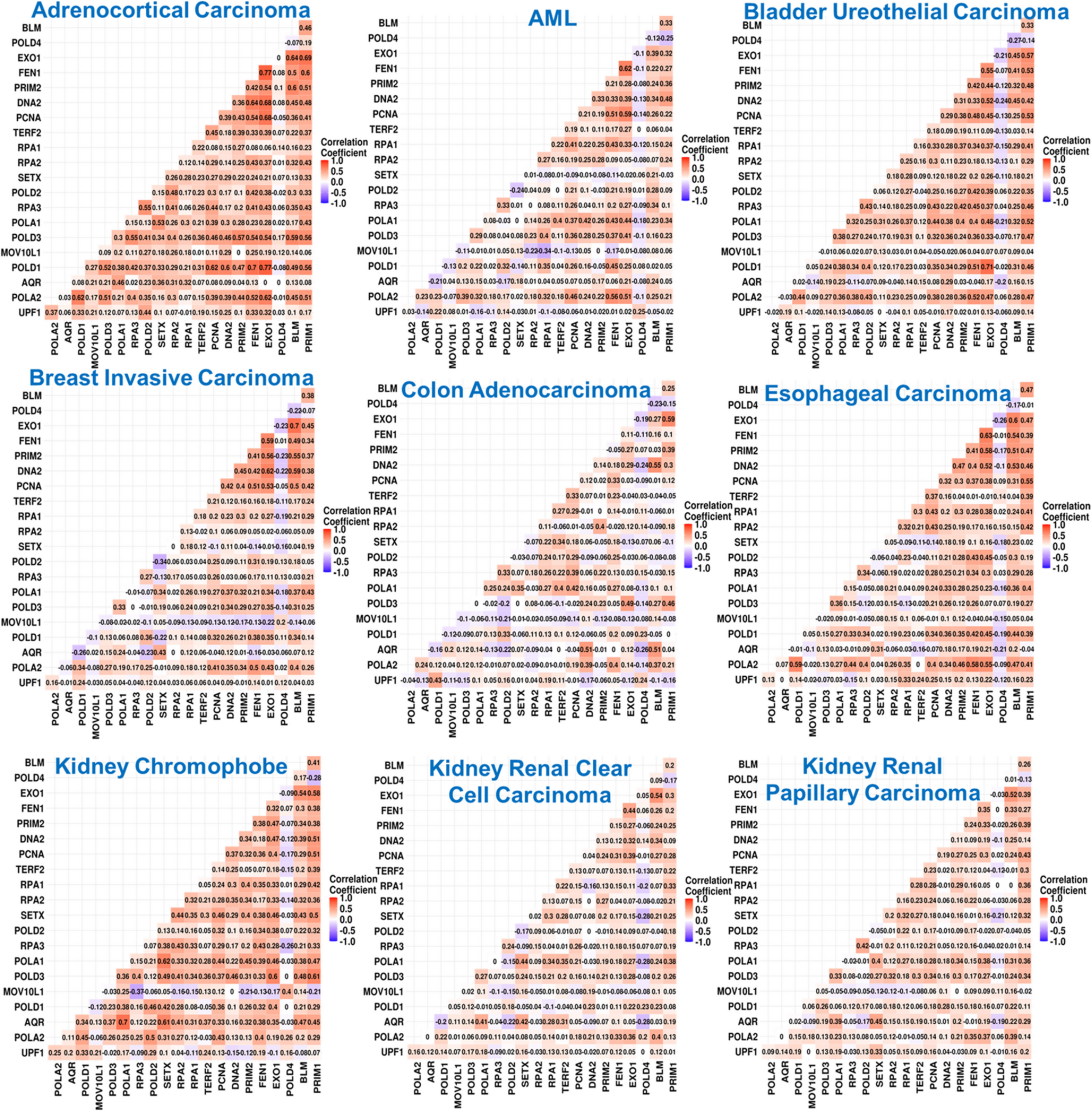


Correct Figure 8
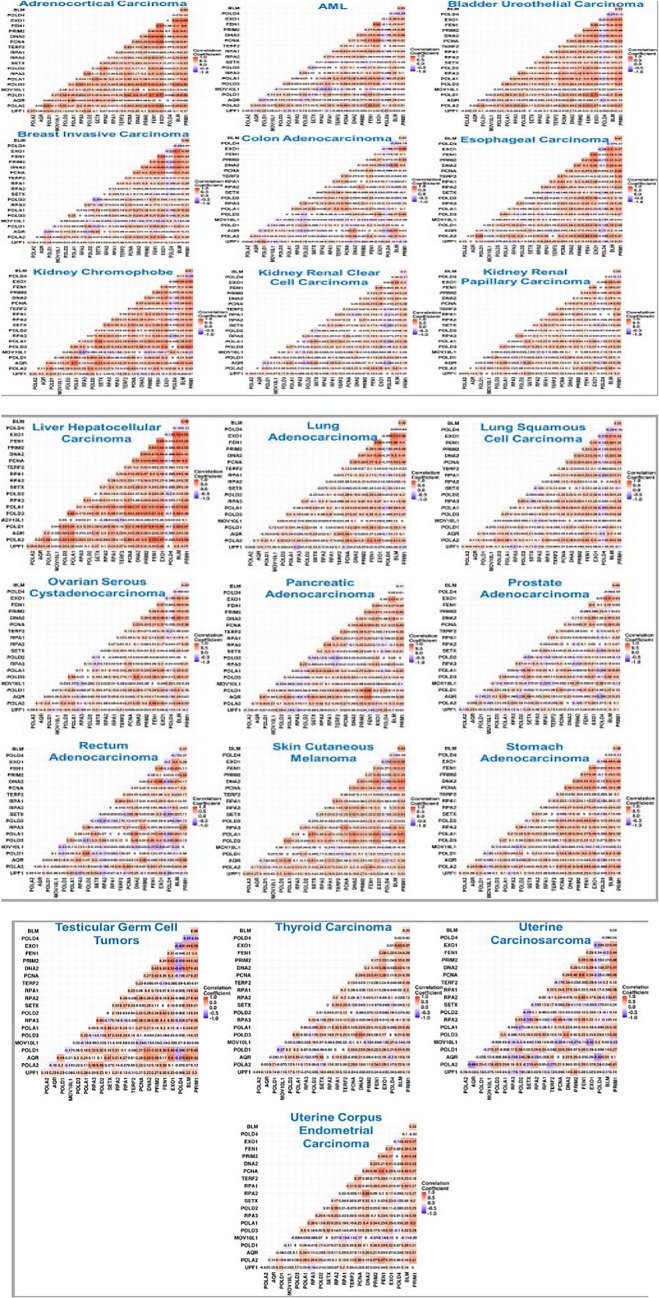


The original article has been corrected.

