# Supplementary material for: Multi-omics analysis positions DNA2 at the interface of genome integrity programs and tumor behavior in pan-cancer
Source: Funct Integr Genomics. 2026 Jun 17;26(1):147. doi: 10.1007/s10142-026-01941-w (PMC13275593; doi:10.1007/s10142-026-01941-w)
Supplement: Supplementary file 1 — Supplementary Material 1 (DOCX 21.0 KB) [file 10142_2026_1941_MOESM1_ESM.docx]

Supplementary 1: Table summarizes survival association results of DNA2 expression across different cancers, indicating whether high or low expression is linked to better or worse overall survival in each cancer type. Significant associations are considered when p≤0.05.
